
JOURNAL INFORMATION
==============================
NLM Title Abbreviation: Can Med Educ J
Journal ID: Can Med Educ J
NLM Journal ID: 101560935
Journal ID: CMEJ

Canadian Medical Education Journal

EISSN: 1923-1202
Publisher: University of Saskatchewan

ARTICLE INFORMATION
==============================
PMCID: PMC7162310
DOI: 10.36834/cmej.70081
Article ID: CMEJ-11-e031
Article version: 1
Subjects: Abstracts

Oral Abstracts

Electronic publication date: 2020 Apr 18
Publication date: 2020 Apr
Volume: 11
Issue: 2
First page: e31
Last page: e154
Copyright: © 2020; 11(2) licensee Synergies Partners
Copyright year: 2020
License: This is an Open Journal Systems article distributed under the terms of the Creative Commons Attribution License (http://creativecommons.org/licenses/by/2.0) which permits unrestricted use, distribution, and reproduction in any medium, provided the original work is properly cited.
License URL: https://creativecommons.org/licenses/by/2.0/

==============================
JOURNAL INFORMATION
==============================
NLM Title Abbreviation: Can Med Educ J
Journal ID: Can Med Educ J
NLM Journal ID: 101560935
Journal ID: CMEJ

Canadian Medical Education Journal

EISSN: 1923-1202
Publisher: University of Saskatchewan

ARTICLE INFORMATION
==============================
Subjects: Oa 1–1

Documenting the process of using electronic health record data to assess residents' performance: Unveiling an Emergency Medicine resident prototype report card

Stefanie Sebok-Syer Stanford University

Adam Dukelow Western University

Lisa Shepherd Western University

Robert Sedran Western University

Allison McConnell Western University

Lorelei Lingard Western University

Electronic publication date: 2020 Apr 18

JOURNAL INFORMATION
==============================
NLM Title Abbreviation: Can Med Educ J
Journal ID: Can Med Educ J
NLM Journal ID: 101560935
Journal ID: CMEJ

Canadian Medical Education Journal

EISSN: 1923-1202
Publisher: University of Saskatchewan

ARTICLE INFORMATION
==============================
Subjects: Oa 1–2

Perceptions of Key Canadian Stakeholders: What are the Philosophies, Principles and Unintended Consequences of CBME?

Kelly Dore McMaster University

Shiphra Ginsburg University of Toronto

Glenn Regehr University of British Columbia

Jonathan Sherbino McMaster University

Electronic publication date: 2020 Apr 18

JOURNAL INFORMATION
==============================
NLM Title Abbreviation: Can Med Educ J
Journal ID: Can Med Educ J
NLM Journal ID: 101560935
Journal ID: CMEJ

Canadian Medical Education Journal

EISSN: 1923-1202
Publisher: University of Saskatchewan

ARTICLE INFORMATION
==============================
Subjects: Oa 1–3

Spotting Potential Opportunities for Teachable moments (SPOT)

Spencer Sample McMaster University

Hussein Al-Rimawi McMaster University

Teresa Chan McMaster University

Electronic publication date: 2020 Apr 18

JOURNAL INFORMATION
==============================
NLM Title Abbreviation: Can Med Educ J
Journal ID: Can Med Educ J
NLM Journal ID: 101560935
Journal ID: CMEJ

Canadian Medical Education Journal

EISSN: 1923-1202
Publisher: University of Saskatchewan

ARTICLE INFORMATION
==============================
Subjects: Oa 1–4

The Shift to Competency Based Medical Education in Canada: A Qualitative Study of Resident Experiences

Leora Branfield Day University of Toronto

Terry Colbourne University of Manitoba

Alex Ng MemorialUniversity of Newfoundland

Franco Rizzuti University of Calgary

Linda Zhou University of British Columbia

Rani Mungroo Resident Doctors of Canada

Allan McDougall Canadian Medical Protective Association

Electronic publication date: 2020 Apr 18

JOURNAL INFORMATION
==============================
NLM Title Abbreviation: Can Med Educ J
Journal ID: Can Med Educ J
NLM Journal ID: 101560935
Journal ID: CMEJ

Canadian Medical Education Journal

EISSN: 1923-1202
Publisher: University of Saskatchewan

ARTICLE INFORMATION
==============================
Subjects: Oa 1–5

Using a Rapid-Cycle Approach to Evaluate Implementation of Competency-Based Medical Education

Stephanie Baxter Queen’s University

Heather Braund Queen’s University

Tessa Hanmore Queen’s University

Nancy Dalgarno Queen’s University

Electronic publication date: 2020 Apr 18

JOURNAL INFORMATION
==============================
NLM Title Abbreviation: Can Med Educ J
Journal ID: Can Med Educ J
NLM Journal ID: 101560935
Journal ID: CMEJ

Canadian Medical Education Journal

EISSN: 1923-1202
Publisher: University of Saskatchewan

ARTICLE INFORMATION
==============================
Subjects: Oa 1–6

Written-based progress testing: a scoping review

Debra Pugh Medical Council of Canada

Vincent Dion Université de Sherbrooke

Ilona Bartman Medical Council of Canada

Claire Touchie Medical Council of Canada

Christina St-Onge Université de Sherbrooke

Electronic publication date: 2020 Apr 18

JOURNAL INFORMATION
==============================
NLM Title Abbreviation: Can Med Educ J
Journal ID: Can Med Educ J
NLM Journal ID: 101560935
Journal ID: CMEJ

Canadian Medical Education Journal

EISSN: 1923-1202
Publisher: University of Saskatchewan

ARTICLE INFORMATION
==============================
Subjects: Oa 2–1

Examining Myths in Assessment: An Opportunity to Advance Trustworthiness in Assessment

Carlos Gomez-Garibello McGill

Maryam Wagner McGill

Valerie Dory

Electronic publication date: 2020 Apr 18

JOURNAL INFORMATION
==============================
NLM Title Abbreviation: Can Med Educ J
Journal ID: Can Med Educ J
NLM Journal ID: 101560935
Journal ID: CMEJ

Canadian Medical Education Journal

EISSN: 1923-1202
Publisher: University of Saskatchewan

ARTICLE INFORMATION
==============================
Subjects: Oa 2–2

Using the College of Family Physicians of Canada's(CFPC) Key Features as core competencies for Family Medicine(FM) Entrustable Professional Activities(EPAs); a mapping and validation project.

Keith Wycliffe-Jones University of Calgary

Jacqueline Hui University of Calgary

Joshua Brochu University of Calgary

Electronic publication date: 2020 Apr 18

JOURNAL INFORMATION
==============================
NLM Title Abbreviation: Can Med Educ J
Journal ID: Can Med Educ J
NLM Journal ID: 101560935
Journal ID: CMEJ

Canadian Medical Education Journal

EISSN: 1923-1202
Publisher: University of Saskatchewan

ARTICLE INFORMATION
==============================
Subjects: Oa 2–3

Sorry to bother you: Requesting and providing clinical support on clinical teaching units

Tristen Gilchrist University of British Columbia

Rose Hatala University of British Columbia

Andrea Gingerich University of British Columbia

Electronic publication date: 2020 Apr 18

JOURNAL INFORMATION
==============================
NLM Title Abbreviation: Can Med Educ J
Journal ID: Can Med Educ J
NLM Journal ID: 101560935
Journal ID: CMEJ

Canadian Medical Education Journal

EISSN: 1923-1202
Publisher: University of Saskatchewan

ARTICLE INFORMATION
==============================
Subjects: Oa 2–4

Bridging Role Conceptualizations to Entrustment in Athletic Therapy

Jeffrey Owen University of Calgary

Mark Lafave Mount Royal University

Michelle Yeo Mount Royal University

Maria Palacios Mackay Universidad San Sebastián

Elizabeth Oddone Paolucci University of Calgary

Electronic publication date: 2020 Apr 18

JOURNAL INFORMATION
==============================
NLM Title Abbreviation: Can Med Educ J
Journal ID: Can Med Educ J
NLM Journal ID: 101560935
Journal ID: CMEJ

Canadian Medical Education Journal

EISSN: 1923-1202
Publisher: University of Saskatchewan

ARTICLE INFORMATION
==============================
Subjects: Oa 2–5

It's a Matter of Trust: Faculty perceptions of an entrustment scale in Family Medicine maternity care assessment

Milena Forte University of Toronto

Natalie Morson University of Toronto

Natasha Mirchandani University of Toronto

Warren Rubenstein University of Toronto

Batya Grundland University of Toronto

Oshan Fernando Western University

Electronic publication date: 2020 Apr 18

JOURNAL INFORMATION
==============================
NLM Title Abbreviation: Can Med Educ J
Journal ID: Can Med Educ J
NLM Journal ID: 101560935
Journal ID: CMEJ

Canadian Medical Education Journal

EISSN: 1923-1202
Publisher: University of Saskatchewan

ARTICLE INFORMATION
==============================
Subjects: Oa 2–6

Pharmacy students use guided reflection and entrustment (EPA) assessments to appraise 'secret' patient/pharmacist encounters in the self-care community workplace

Debra Sibbald University of Toronto

Electronic publication date: 2020 Apr 18

JOURNAL INFORMATION
==============================
NLM Title Abbreviation: Can Med Educ J
Journal ID: Can Med Educ J
NLM Journal ID: 101560935
Journal ID: CMEJ

Canadian Medical Education Journal

EISSN: 1923-1202
Publisher: University of Saskatchewan

ARTICLE INFORMATION
==============================
Subjects: Oa 3–1

Developmental Progress Assessment: A Scoping Review

Christina St-Onge Université de Sherbrooke

Élise Vachon Lachiver Université de Sherbrooke

Serge Langevin Université de Sherbrooke

Elisabeth Boileau Université de Sherbrooke

Frédéric Bernier Université de Sherbrooke

Aliki Thomas McGill

Electronic publication date: 2020 Apr 18

JOURNAL INFORMATION
==============================
NLM Title Abbreviation: Can Med Educ J
Journal ID: Can Med Educ J
NLM Journal ID: 101560935
Journal ID: CMEJ

Canadian Medical Education Journal

EISSN: 1923-1202
Publisher: University of Saskatchewan

ARTICLE INFORMATION
==============================
Subjects: Oa 3–2

When we talk about “Assessment for learning” what does it imply?

Elise Vachon Lachiver Université de Sherbrooke

Maude Lemay Bishop's University

Christina St-Onge Université de Sherbrooke

Aliki Thomas McGill

Meghan McConnell University of Ottawa

Electronic publication date: 2020 Apr 18

JOURNAL INFORMATION
==============================
NLM Title Abbreviation: Can Med Educ J
Journal ID: Can Med Educ J
NLM Journal ID: 101560935
Journal ID: CMEJ

Canadian Medical Education Journal

EISSN: 1923-1202
Publisher: University of Saskatchewan

ARTICLE INFORMATION
==============================
Subjects: Oa 3–3

The Representative Model for Educational Impact of Assessment Program on Medical Students' Learning

Mohammad Jalili Tehran University of Medical Sciences

Azadeh Kordestani Moghadam

Hamidreza Khankeh

Electronic publication date: 2020 Apr 18

JOURNAL INFORMATION
==============================
NLM Title Abbreviation: Can Med Educ J
Journal ID: Can Med Educ J
NLM Journal ID: 101560935
Journal ID: CMEJ

Canadian Medical Education Journal

EISSN: 1923-1202
Publisher: University of Saskatchewan

ARTICLE INFORMATION
==============================
Subjects: Oa 3–4

Validation of a grid to document the quality of structured reflection when implemented as a learning strategy at the UGME level

Élise Vachon Lachiver Université de Sherbrooke

Martine Chamberland Université de Sherbrooke

Jean Setrakian Université de Sherbrooke

Mélanie Marceau Université de Sherbrooke

Julie Ouellet Université de Sherbrooke

Christian Campagna Université de Sherbrooke

Linda Bergeron Université de Sherbrooke

Christina St-Onge Université de Sherbrooke

Electronic publication date: 2020 Apr 18

JOURNAL INFORMATION
==============================
NLM Title Abbreviation: Can Med Educ J
Journal ID: Can Med Educ J
NLM Journal ID: 101560935
Journal ID: CMEJ

Canadian Medical Education Journal

EISSN: 1923-1202
Publisher: University of Saskatchewan

ARTICLE INFORMATION
==============================
Subjects: Oa 3–5

Développement et validation d'une fiche de rétroaction francophone pour l'observation directe des résidents dans les programmes de médecine familiale au Canada - phase 1 : étude de besoins et validation de contenu

Miriam Lacasse Université Laval

Luc Côté Université Laval

Gabrielle Hogue Université Laval

Pascal Lalancette Université Laval

Jean-Sébastien Renaud Université Laval

Christian Rheault Université Laval

Marion Dove McGill

Marie-Pierre Codsi Université de Montréal

Lyne Pitre University of Ottawa

Luce Pélissier-Simard Université de Sherbrooke

Electronic publication date: 2020 Apr 18

JOURNAL INFORMATION
==============================
NLM Title Abbreviation: Can Med Educ J
Journal ID: Can Med Educ J
NLM Journal ID: 101560935
Journal ID: CMEJ

Canadian Medical Education Journal

EISSN: 1923-1202
Publisher: University of Saskatchewan

ARTICLE INFORMATION
==============================
Subjects: Oa 3–6

The Role of Previously Undocumented Data in the Assessment of Medical Trainees in Clinical Competency Committees

Jennifer Tam University of British Columbia

Glenn Regehr University of British Columbia

Anupma Wadhwa University of Toronto

Maria Athina (Tina) Martimianakis University of Toronto

Oshan Fernando University of Toronto

Electronic publication date: 2020 Apr 18

JOURNAL INFORMATION
==============================
NLM Title Abbreviation: Can Med Educ J
Journal ID: Can Med Educ J
NLM Journal ID: 101560935
Journal ID: CMEJ

Canadian Medical Education Journal

EISSN: 1923-1202
Publisher: University of Saskatchewan

ARTICLE INFORMATION
==============================
Subjects: Oa 4–1

Health Humanities and Social Accountability in Action: Working Collaboratively across Disciplines and Communities

Pamela Brett-MacLean University of Alberta

Hollis Lai University of Alberta

Tracey Hillier University of Alberta

Helly Goez University of Alberta

Helly Goez University of Alberta

Electronic publication date: 2020 Apr 18

JOURNAL INFORMATION
==============================
NLM Title Abbreviation: Can Med Educ J
Journal ID: Can Med Educ J
NLM Journal ID: 101560935
Journal ID: CMEJ

Canadian Medical Education Journal

EISSN: 1923-1202
Publisher: University of Saskatchewan

ARTICLE INFORMATION
==============================
Subjects: Oa 4–2

The competency profile of the socially responsible health professional: citizens' points of view

Emmanuelle Careau Université Laval

Marie-Claire Bérubé Université Laval

Émélie Provost Université Laval

Julien Poitras Université Laval

Electronic publication date: 2020 Apr 18

JOURNAL INFORMATION
==============================
NLM Title Abbreviation: Can Med Educ J
Journal ID: Can Med Educ J
NLM Journal ID: 101560935
Journal ID: CMEJ

Canadian Medical Education Journal

EISSN: 1923-1202
Publisher: University of Saskatchewan

ARTICLE INFORMATION
==============================
Subjects: Oa 4–3

How to follow a more « socially accountable » institutional strategic planning: the Université Laval's Faculté de médecine experience

Emmanuelle Careau Université Laval

Geneviève Bhérer Université Laval

Marie-Chantal Denis Université Laval

Caroline Dugal Université Laval

Patrice Lemay Université Laval

Julien Poitras Université Laval

Electronic publication date: 2020 Apr 18

JOURNAL INFORMATION
==============================
NLM Title Abbreviation: Can Med Educ J
Journal ID: Can Med Educ J
NLM Journal ID: 101560935
Journal ID: CMEJ

Canadian Medical Education Journal

EISSN: 1923-1202
Publisher: University of Saskatchewan

ARTICLE INFORMATION
==============================
Subjects: Oa 4–4

Evaluating the scope of social accountability in medical student summer research projects

Desiree Naude University of Saskatchewan

Erin Walling University of Saskatchewan

Marcel D'Eon University of Saskatchewan

Electronic publication date: 2020 Apr 18

JOURNAL INFORMATION
==============================
NLM Title Abbreviation: Can Med Educ J
Journal ID: Can Med Educ J
NLM Journal ID: 101560935
Journal ID: CMEJ

Canadian Medical Education Journal

EISSN: 1923-1202
Publisher: University of Saskatchewan

ARTICLE INFORMATION
==============================
Subjects: Oa 4–5

Developing a Social Accountability Praxis: How Students Perspectives Change Across Undergraduate Medical Education

Erin Cameron Northern Ontario School of Medicine

Jacqueline Harvey Northern Ontario School of Medicine

Holly Fleming Northern Ontario School of Medicine

Electronic publication date: 2020 Apr 18

JOURNAL INFORMATION
==============================
NLM Title Abbreviation: Can Med Educ J
Journal ID: Can Med Educ J
NLM Journal ID: 101560935
Journal ID: CMEJ

Canadian Medical Education Journal

EISSN: 1923-1202
Publisher: University of Saskatchewan

ARTICLE INFORMATION
==============================
Subjects: Oa 4–6

The distribution of available medical school opportunities in Canada by province of residence

Lawrence Grierson McMaster University

Meredith Vanstone McMaster University

Electronic publication date: 2020 Apr 18

JOURNAL INFORMATION
==============================
NLM Title Abbreviation: Can Med Educ J
Journal ID: Can Med Educ J
NLM Journal ID: 101560935
Journal ID: CMEJ

Canadian Medical Education Journal

EISSN: 1923-1202
Publisher: University of Saskatchewan

ARTICLE INFORMATION
==============================
Subjects: Oa 5–1

The Threads Among Us: Teaching Interprofessional Empathy as an Antidote to Incivility

Ellen M. Friedman Baylor College of Medicine

Jordan Shapiro Baylor Collge of Medicine

Electronic publication date: 2020 Apr 18

JOURNAL INFORMATION
==============================
NLM Title Abbreviation: Can Med Educ J
Journal ID: Can Med Educ J
NLM Journal ID: 101560935
Journal ID: CMEJ

Canadian Medical Education Journal

EISSN: 1923-1202
Publisher: University of Saskatchewan

ARTICLE INFORMATION
==============================
Subjects: Oa 5–2

Getting them to the table: Engaging inter-professional team members to talk about their workplace practices

Lori Nemoy St. Michael's Hospital, Unity Health Toronto

Kristen Daly St. Michael's Hospital, Unity Health Toronto

Christine Léger St. Michael's Hospital, Unity Health Toronto

Nazanin Khodadoust St. Michael's Hospital, Unity Health Toronto

Douglas Campbell St. Michael's Hospital, Unity Health Toronto

Ryan Brydges St. Michael's Hospital, Unity Health Toronto

Electronic publication date: 2020 Apr 18

JOURNAL INFORMATION
==============================
NLM Title Abbreviation: Can Med Educ J
Journal ID: Can Med Educ J
NLM Journal ID: 101560935
Journal ID: CMEJ

Canadian Medical Education Journal

EISSN: 1923-1202
Publisher: University of Saskatchewan

ARTICLE INFORMATION
==============================
Subjects: Oa 5–3

Exploring how implicit biases influence inter-professional collaboration using a socio-material framework

Javeed Sukhera Western University

Kaitlyn Bertram Western University

Shawn Hendrikx Western University

Margaret Chisolm Johns Hopkins

Juliette Perzhinsky Western Michigan University

Erin Kennedy Western University

Lorelei Lingard Western University

Mark Goldszmidt Western University

Electronic publication date: 2020 Apr 18

JOURNAL INFORMATION
==============================
NLM Title Abbreviation: Can Med Educ J
Journal ID: Can Med Educ J
NLM Journal ID: 101560935
Journal ID: CMEJ

Canadian Medical Education Journal

EISSN: 1923-1202
Publisher: University of Saskatchewan

ARTICLE INFORMATION
==============================
Subjects: Oa 5–4

Needs assessment study to build interprofessional in situ simulation training in non-technical skills: improving quality of care and patient safety during acute clinical adverse events.

Ahmed Moussa Université de Montréal

Claude-Julie Bourque Université de Montréal

Nathalie Loye Université de Montréal

Audrey Larone Juneau CHU Sainte-Justine

Evelyne Wassef Université de Montréal

Ahmed Moussa Université de Montréal

Emilie Dumont CHU Sainte-Justine

Michael-Andrew Assaad Université de Montréal

Electronic publication date: 2020 Apr 18

JOURNAL INFORMATION
==============================
NLM Title Abbreviation: Can Med Educ J
Journal ID: Can Med Educ J
NLM Journal ID: 101560935
Journal ID: CMEJ

Canadian Medical Education Journal

EISSN: 1923-1202
Publisher: University of Saskatchewan

ARTICLE INFORMATION
==============================
Subjects: Oa 5–5

“Now I know it, Now I'll do it, I'll work on that”: the nature of learning after team based simulation

Farhana Shariff University of British Columbia

Glenn Regehr University of British Columbia

Rose Hatala University of British Columbia

Electronic publication date: 2020 Apr 18

JOURNAL INFORMATION
==============================
NLM Title Abbreviation: Can Med Educ J
Journal ID: Can Med Educ J
NLM Journal ID: 101560935
Journal ID: CMEJ

Canadian Medical Education Journal

EISSN: 1923-1202
Publisher: University of Saskatchewan

ARTICLE INFORMATION
==============================
Subjects: Oa 5–6

Evaluating interest in a Novel Educational Program for Integrated Team-Based Care of Patients with both Physical and Mental Illness across different educational platforms

Alison Freeland University of Toronto

Sanjeev Sockalingam University of Toronto

David wiljer University of Toronto

Alicia Lozon University of Toronto

Gurpreet Grewal Trillium Health Partners

Electronic publication date: 2020 Apr 18

JOURNAL INFORMATION
==============================
NLM Title Abbreviation: Can Med Educ J
Journal ID: Can Med Educ J
NLM Journal ID: 101560935
Journal ID: CMEJ

Canadian Medical Education Journal

EISSN: 1923-1202
Publisher: University of Saskatchewan

ARTICLE INFORMATION
==============================
Subjects: Oa 6–1

Evaluation of a Deteriorating Patient Simulation to Teach Integrated Physical and Mental Health Care in Undergraduate Medical Education

Fadi Gorgi University of Toronto

Zarah Chaudhary Wilson Centre, University Health Network and University of Toronto

Carla Garcia University of Toronto

Maria Mylopoulos Wilson Centre, University Health Network and University of Toronto

Sanjeev Sockalingam University of Toronto

Electronic publication date: 2020 Apr 18

JOURNAL INFORMATION
==============================
NLM Title Abbreviation: Can Med Educ J
Journal ID: Can Med Educ J
NLM Journal ID: 101560935
Journal ID: CMEJ

Canadian Medical Education Journal

EISSN: 1923-1202
Publisher: University of Saskatchewan

ARTICLE INFORMATION
==============================
Subjects: Oa 6–2

Realistic Preparation for Going Global: Pre-departure Training Driven by Role-Playing Case Study Simulations

Jennifer Carpenter Queen’s University

Guy Sheahan Queen’s University

Eleftherios Soleas Queen’s University

Mikaila De sousa Queen’s University

Elizabeth Matzinger Queen’s University

Nicholas Cofie Queen’s University

Electronic publication date: 2020 Apr 18

JOURNAL INFORMATION
==============================
NLM Title Abbreviation: Can Med Educ J
Journal ID: Can Med Educ J
NLM Journal ID: 101560935
Journal ID: CMEJ

Canadian Medical Education Journal

EISSN: 1923-1202
Publisher: University of Saskatchewan

ARTICLE INFORMATION
==============================
Subjects: Oa 6–3

The invisible work of Cadaver Based Simulation: An ethnography

Victoria Luong Dalhousie University

Paula Cameron Dalhousie University

Anna MacLeod Dalhousie University

Olga Kits Dalhousie University

Lucy Patrick Dalhousie University

George Kovacs Dalhousie University

Jonathan Tummons Durham University

Molly Fredeen Dalhousie University

Victoria Luong Dalhousie University

Electronic publication date: 2020 Apr 18

JOURNAL INFORMATION
==============================
NLM Title Abbreviation: Can Med Educ J
Journal ID: Can Med Educ J
NLM Journal ID: 101560935
Journal ID: CMEJ

Canadian Medical Education Journal

EISSN: 1923-1202
Publisher: University of Saskatchewan

ARTICLE INFORMATION
==============================
Subjects: Oa 6–4

Sharing Experiential Knowledge to Enhance Collaborative Practice in the Emergency Room

Nicolas Fernandez Université de Montréal

Electronic publication date: 2020 Apr 18

JOURNAL INFORMATION
==============================
NLM Title Abbreviation: Can Med Educ J
Journal ID: Can Med Educ J
NLM Journal ID: 101560935
Journal ID: CMEJ

Canadian Medical Education Journal

EISSN: 1923-1202
Publisher: University of Saskatchewan

ARTICLE INFORMATION
==============================
Subjects: Oa 6–5

“She's a manikin; she won't mind”: A sociomaterial look at patient centredness in manikin-based simulation

Paula Cameron Dalhousie University

Molly Fredeen Dalhousie University

Anna MacLeod Dalhousie University

Victoria Luong Dalhousie University

Rola Ajjawi Deakin University, Melbourne AUS

Jonathan Tummons Durham University

Olga Kits Dalhousie University

Marti Cleveland-Innes Athabasca

Electronic publication date: 2020 Apr 18

JOURNAL INFORMATION
==============================
NLM Title Abbreviation: Can Med Educ J
Journal ID: Can Med Educ J
NLM Journal ID: 101560935
Journal ID: CMEJ

Canadian Medical Education Journal

EISSN: 1923-1202
Publisher: University of Saskatchewan

ARTICLE INFORMATION
==============================
Subjects: Oa 6–6

Engaging Learner Perspectives: A realist analysis of learner perceptions of safety in high stakes simulations

Joanna Rankin University of Calgary

Rachel Grimminck University of Calgary

Paige Durling University of Calgary

Amber Barlow University of Calgary

Jihane Henni University of Calgary

Dean Mrozowich University of Calgary

Electronic publication date: 2020 Apr 18

JOURNAL INFORMATION
==============================
NLM Title Abbreviation: Can Med Educ J
Journal ID: Can Med Educ J
NLM Journal ID: 101560935
Journal ID: CMEJ

Canadian Medical Education Journal

EISSN: 1923-1202
Publisher: University of Saskatchewan

ARTICLE INFORMATION
==============================
Subjects: Ob 1–2

A stitch in time saves nine: Rethinking curricular organization in response to student burn out

Liam Dowling Queen’s University

Andrea Guerin Queen’s University

Lindsay Davidson Queen’s University

Electronic publication date: 2020 Apr 18

JOURNAL INFORMATION
==============================
NLM Title Abbreviation: Can Med Educ J
Journal ID: Can Med Educ J
NLM Journal ID: 101560935
Journal ID: CMEJ

Canadian Medical Education Journal

EISSN: 1923-1202
Publisher: University of Saskatchewan

ARTICLE INFORMATION
==============================
Subjects: Ob 1–3

Development and Implementation of a Comprehensive Peer Support Program for Medical Students at the University of Ottawa

Kelsey Mongrain University of Ottawa

Kay-Anne Haykal University of Ottawa

Electronic publication date: 2020 Apr 18

JOURNAL INFORMATION
==============================
NLM Title Abbreviation: Can Med Educ J
Journal ID: Can Med Educ J
NLM Journal ID: 101560935
Journal ID: CMEJ

Canadian Medical Education Journal

EISSN: 1923-1202
Publisher: University of Saskatchewan

ARTICLE INFORMATION
==============================
Subjects: Ob 1–4

Cultural Responsiveness Training in Post Graduate Medical Education

James Barton University of Saskatchewan

Anurag Saxena University of Saskatchewan

Stacey Lovo University of Saskatchewan

Veronica McKinney University of Saskatchewan

Electronic publication date: 2020 Apr 18

JOURNAL INFORMATION
==============================
NLM Title Abbreviation: Can Med Educ J
Journal ID: Can Med Educ J
NLM Journal ID: 101560935
Journal ID: CMEJ

Canadian Medical Education Journal

EISSN: 1923-1202
Publisher: University of Saskatchewan

ARTICLE INFORMATION
==============================
Subjects: Ob 1–5

Prevalence of resident burnout remains unchanged worldwide despite efforts in the last 20 years: are we missing the point?

Leen Naji McMaster University

Zahra Sohani McGill

Electronic publication date: 2020 Apr 18

JOURNAL INFORMATION
==============================
NLM Title Abbreviation: Can Med Educ J
Journal ID: Can Med Educ J
NLM Journal ID: 101560935
Journal ID: CMEJ

Canadian Medical Education Journal

EISSN: 1923-1202
Publisher: University of Saskatchewan

ARTICLE INFORMATION
==============================
Subjects: Ob 1–6

Getting into the Zone: More to Cognitive Flow than Cognition Alone?

Sydney McQueen University of Toronto

Aidan McParland University of Toronto

Melanie Hammond Mobilio University of Toronto

Carol-anne Moulton University of Toronto

Electronic publication date: 2020 Apr 18

JOURNAL INFORMATION
==============================
NLM Title Abbreviation: Can Med Educ J
Journal ID: Can Med Educ J
NLM Journal ID: 101560935
Journal ID: CMEJ

Canadian Medical Education Journal

EISSN: 1923-1202
Publisher: University of Saskatchewan

ARTICLE INFORMATION
==============================
Subjects: Ob 1–7

“How are you?” Physicians Describe Meaningful Peer Support

Tandi Wilkinson University of British Columbia

Rola Ajjawi Deakin University, Melbourne Australia

Shireen Mansouri University of Calgary

Electronic publication date: 2020 Apr 18

JOURNAL INFORMATION
==============================
NLM Title Abbreviation: Can Med Educ J
Journal ID: Can Med Educ J
NLM Journal ID: 101560935
Journal ID: CMEJ

Canadian Medical Education Journal

EISSN: 1923-1202
Publisher: University of Saskatchewan

ARTICLE INFORMATION
==============================
Subjects: Ob 2–1

In Anticipation of CBD: Building an Integrative, Skills-Based Transition to Practice Curriculum in Psychiatry

Natasha Snelgrove McMaster University

Sheila Harms McMaster University

JoAnn Corey McMaster University

Electronic publication date: 2020 Apr 18

JOURNAL INFORMATION
==============================
NLM Title Abbreviation: Can Med Educ J
Journal ID: Can Med Educ J
NLM Journal ID: 101560935
Journal ID: CMEJ

Canadian Medical Education Journal

EISSN: 1923-1202
Publisher: University of Saskatchewan

ARTICLE INFORMATION
==============================
Subjects: Ob 2–2

Static in the line or a full disconnect? Exploring resident perceptions and interpretations of initial competency based medical education (CBME) implementation compared to the core components of CBME

Shivani Upadhyaya University of Alberta

Marghalara Rashid University of Alberta

Andrea Davila-Cervantes University of Alberta

Anna Oswald University of Alberta

Electronic publication date: 2020 Apr 18

JOURNAL INFORMATION
==============================
NLM Title Abbreviation: Can Med Educ J
Journal ID: Can Med Educ J
NLM Journal ID: 101560935
Journal ID: CMEJ

Canadian Medical Education Journal

EISSN: 1923-1202
Publisher: University of Saskatchewan

ARTICLE INFORMATION
==============================
Subjects: Ob 2–3

Exploring Residents' Perspectives of Competency-Based Medical Education Across Canada

Vivesh Patel Queen’s University

Stephen Mann Queen’s University

Heather Braund Queen’s University

Nancy Dalgarno Queen’s University

Electronic publication date: 2020 Apr 18

JOURNAL INFORMATION
==============================
NLM Title Abbreviation: Can Med Educ J
Journal ID: Can Med Educ J
NLM Journal ID: 101560935
Journal ID: CMEJ

Canadian Medical Education Journal

EISSN: 1923-1202
Publisher: University of Saskatchewan

ARTICLE INFORMATION
==============================
Subjects: Ob 2–6

Low-stakes progress test results are excellent predictors for success at the Medical Council of Canada Qualifying Examination part I.

Margaret Henri Université de Montréal

Robert Gagnon Université de Montréal

François Gobeil Université de Montréal

Geneviève Grégoire Université de Montréal

Electronic publication date: 2020 Apr 18

JOURNAL INFORMATION
==============================
NLM Title Abbreviation: Can Med Educ J
Journal ID: Can Med Educ J
NLM Journal ID: 101560935
Journal ID: CMEJ

Canadian Medical Education Journal

EISSN: 1923-1202
Publisher: University of Saskatchewan

ARTICLE INFORMATION
==============================
Subjects: Ob 3–1

Bridging the Gap: Improving CASPer Test Confidence and Competency for Underrepresented Minorities in Medicine through Interactive Peer-assisted Learning

Lolade Shipeolu University of Ottawa

Johanne Matthieu University of Ottawa

Farhan Mahmood University of Ottawa

Ike Okafor University of Toronto

Electronic publication date: 2020 Apr 18

JOURNAL INFORMATION
==============================
NLM Title Abbreviation: Can Med Educ J
Journal ID: Can Med Educ J
NLM Journal ID: 101560935
Journal ID: CMEJ

Canadian Medical Education Journal

EISSN: 1923-1202
Publisher: University of Saskatchewan

ARTICLE INFORMATION
==============================
Subjects: Ob 3–2

O Negative Blood - Experiences from a Medical Education Teaching Rotation

Amanda Jones University of British Columbia

Alasdair Nazerali Maitland University of British Columbia

Samantha Stasiuk University of British Columbia

Amil Shah University of British Columbia

Reed Holden University of British Columbia

Electronic publication date: 2020 Apr 18

JOURNAL INFORMATION
==============================
NLM Title Abbreviation: Can Med Educ J
Journal ID: Can Med Educ J
NLM Journal ID: 101560935
Journal ID: CMEJ

Canadian Medical Education Journal

EISSN: 1923-1202
Publisher: University of Saskatchewan

ARTICLE INFORMATION
==============================
Subjects: Ob 3–3

R2C2 for “in the moment” feedback and coaching

Heather Armson University of Calgary

Rachelle Lee-Krueger University of Calgary

Karen Könings Maastricht University

Amanda Roze des Ordons University of Calgary

Jessica Trier Queen’s University

Marygrace Zetkulik Hackensack Meridian School of Medicine

Jocelyn Lockyer University of Calgary

Joan Sargeant Dalhousie University

Electronic publication date: 2020 Apr 18

JOURNAL INFORMATION
==============================
NLM Title Abbreviation: Can Med Educ J
Journal ID: Can Med Educ J
NLM Journal ID: 101560935
Journal ID: CMEJ

Canadian Medical Education Journal

EISSN: 1923-1202
Publisher: University of Saskatchewan

ARTICLE INFORMATION
==============================
Subjects: Ob 3–4

Coaching: a state-of-the-art review

Victoria McKinnon McMaster University

Cindy Tran McMaster University

Jennifer Zering McMaster University

Ranil Sonnadara McMaster University

Electronic publication date: 2020 Apr 18

JOURNAL INFORMATION
==============================
NLM Title Abbreviation: Can Med Educ J
Journal ID: Can Med Educ J
NLM Journal ID: 101560935
Journal ID: CMEJ

Canadian Medical Education Journal

EISSN: 1923-1202
Publisher: University of Saskatchewan

ARTICLE INFORMATION
==============================
Subjects: Ob 3–5

A Pan-Canadian Evaluation of the College of Family Medicine Canada's Fundamental Teaching Activities Framework

Douglas Archibald University of Ottawa

Dianne Delva Queen’s University

Rachelle Lee-Krueger University of Ottawa

Viola Antao University of Toronto

Cheri Bethune Memorial – University of Newfoundland

Catherine Giroux University of Ottawa

Katherine Moreau University of Ottawa

Electronic publication date: 2020 Apr 18

JOURNAL INFORMATION
==============================
NLM Title Abbreviation: Can Med Educ J
Journal ID: Can Med Educ J
NLM Journal ID: 101560935
Journal ID: CMEJ

Canadian Medical Education Journal

EISSN: 1923-1202
Publisher: University of Saskatchewan

ARTICLE INFORMATION
==============================
Subjects: Ob 3–6

The Diversity Mentorship Program: a Model for Effective Equity-based Mentorship

Imaan Javeed University of Toronto

Anita Balakrishna University of Toronto

Stephanie Zhou University of Toronto

Lisa Robinson University of Toronto

Electronic publication date: 2020 Apr 18

JOURNAL INFORMATION
==============================
NLM Title Abbreviation: Can Med Educ J
Journal ID: Can Med Educ J
NLM Journal ID: 101560935
Journal ID: CMEJ

Canadian Medical Education Journal

EISSN: 1923-1202
Publisher: University of Saskatchewan

ARTICLE INFORMATION
==============================
Subjects: Ob 4–1

Integrating the Humanities to Enhance Clinical Reasoning

Wendy A Stewart Dalhousie University

Mark Gilbert Dalhousie University

Pat Croskerry Dalhousie University

Stephen Miller Dalhousie University

Laura Thomas Dalhousie University

Electronic publication date: 2020 Apr 18

JOURNAL INFORMATION
==============================
NLM Title Abbreviation: Can Med Educ J
Journal ID: Can Med Educ J
NLM Journal ID: 101560935
Journal ID: CMEJ

Canadian Medical Education Journal

EISSN: 1923-1202
Publisher: University of Saskatchewan

ARTICLE INFORMATION
==============================
Subjects: Ob 4–2

Learning by Concept or Learning by Example: An Experimental Study of Teaching Bayesian Diagnosis

Jonathan Sherbino McMaster University

John E. Brush Eastern Virginia Medical School

Mark Lee McMaster University

Judith Taylor-Fishwick Eastern Virginia Medical School

Geoffrey Norman McMaster University

Electronic publication date: 2020 Apr 18

JOURNAL INFORMATION
==============================
NLM Title Abbreviation: Can Med Educ J
Journal ID: Can Med Educ J
NLM Journal ID: 101560935
Journal ID: CMEJ

Canadian Medical Education Journal

EISSN: 1923-1202
Publisher: University of Saskatchewan

ARTICLE INFORMATION
==============================
Subjects: Ob 4–3

Incidentally induced anxiety and clinical reasoning in medical students

Jonathan Misskey University of British Columbia

Kevin Eva University of British Columbia

Electronic publication date: 2020 Apr 18

JOURNAL INFORMATION
==============================
NLM Title Abbreviation: Can Med Educ J
Journal ID: Can Med Educ J
NLM Journal ID: 101560935
Journal ID: CMEJ

Canadian Medical Education Journal

EISSN: 1923-1202
Publisher: University of Saskatchewan

ARTICLE INFORMATION
==============================
Subjects: Ob 4–4

The definition(s) of clinical reasoning

Meredith Young McGill

Aliki Thomas McGill

Ana Da Silva Swansea University

Stuart Lubarksy McGill

Larry Gruppen University of Michigan

David Gordon Duke University

Valerie Dory McGill

Temple Ratcliffe University of Texas Healch Sciences Centre San Antonio

Tiffany Ballard University of Michigan

Joseph Rencic Tufts Medical Centre

Lambert Schuwirth Flinders University

Steven Durning Uniformed Services University for the Health Sciences

Electronic publication date: 2020 Apr 18

JOURNAL INFORMATION
==============================
NLM Title Abbreviation: Can Med Educ J
Journal ID: Can Med Educ J
NLM Journal ID: 101560935
Journal ID: CMEJ

Canadian Medical Education Journal

EISSN: 1923-1202
Publisher: University of Saskatchewan

ARTICLE INFORMATION
==============================
Subjects: Ob 4–5

In Support of Meaningful Assessment and Feedback: A study of Reasoning Tasks Used in Ambulatory Case Reviews

Azin Ahrari University of British Columbia

Jacqueline Torti Western University

Kamilah Abdur-Rashid Western University

Mark Goldszmidt Western University

Electronic publication date: 2020 Apr 18

JOURNAL INFORMATION
==============================
NLM Title Abbreviation: Can Med Educ J
Journal ID: Can Med Educ J
NLM Journal ID: 101560935
Journal ID: CMEJ

Canadian Medical Education Journal

EISSN: 1923-1202
Publisher: University of Saskatchewan

ARTICLE INFORMATION
==============================
Subjects: Ob 4–6

Expert reasoning in the context of ill-structured clinical problems: Exploring the experiences and sources of 'comfort with uncertainty'

Jonathan Ilgen University of Washington

Judith Bowen Washington State University

Pim Teunissen Maastricht University

Anique de Bruin Maastricht University

Glenn Regehr University of British Columbia

Electronic publication date: 2020 Apr 18

JOURNAL INFORMATION
==============================
NLM Title Abbreviation: Can Med Educ J
Journal ID: Can Med Educ J
NLM Journal ID: 101560935
Journal ID: CMEJ

Canadian Medical Education Journal

EISSN: 1923-1202
Publisher: University of Saskatchewan

ARTICLE INFORMATION
==============================
Subjects: Ob 5–1

Evaluating the University of Toronto Medical School's Black Student Application Program (BSAP): Building evidence for a process of community support and outcomes of positive change

Traci Cook University of Toronto

Electronic publication date: 2020 Apr 18

JOURNAL INFORMATION
==============================
NLM Title Abbreviation: Can Med Educ J
Journal ID: Can Med Educ J
NLM Journal ID: 101560935
Journal ID: CMEJ

Canadian Medical Education Journal

EISSN: 1923-1202
Publisher: University of Saskatchewan

ARTICLE INFORMATION
==============================
Subjects: Ob 5–2

Experiences of 'First in Family' Medical Students

Sarah Wright University of Toronto

Victoria Boyd University of Toronto

Nicole Woods University of Toronto

Malika Sharma University of Toronto

Lisa Richardson University of Toronto

Ryan Giroux University of Toronto

Ike Okafor University of Toronto

Maria Mylopoulos University of Toronto

Electronic publication date: 2020 Apr 18

JOURNAL INFORMATION
==============================
NLM Title Abbreviation: Can Med Educ J
Journal ID: Can Med Educ J
NLM Journal ID: 101560935
Journal ID: CMEJ

Canadian Medical Education Journal

EISSN: 1923-1202
Publisher: University of Saskatchewan

ARTICLE INFORMATION
==============================
Subjects: Ob 5–4

No resident left behind: Establishing the need for targeted mentorship for underrepresented residents in Canada

Sung Min (Steven) Cho University of Toronto

Sung Min (Steven) Cho University of Toronto

Anita Balakrishna University of Toronto

Mariela Ruetalo University of Toronto

Lisa Robinson University of Toronto

Glen Bandiera University of Toronto

Electronic publication date: 2020 Apr 18

JOURNAL INFORMATION
==============================
NLM Title Abbreviation: Can Med Educ J
Journal ID: Can Med Educ J
NLM Journal ID: 101560935
Journal ID: CMEJ

Canadian Medical Education Journal

EISSN: 1923-1202
Publisher: University of Saskatchewan

ARTICLE INFORMATION
==============================
Subjects: Ob 5–5

Perceptions and experiences of gender equity in an academic internal medicine department

Shannon Ruzycki University of Calgary

Allison Brown University of Calgary

Aleem Bharwani University of Calgary

Georgina Freeman University of Calgary

Electronic publication date: 2020 Apr 18

JOURNAL INFORMATION
==============================
NLM Title Abbreviation: Can Med Educ J
Journal ID: Can Med Educ J
NLM Journal ID: 101560935
Journal ID: CMEJ

Canadian Medical Education Journal

EISSN: 1923-1202
Publisher: University of Saskatchewan

ARTICLE INFORMATION
==============================
Subjects: Ob 5–6

Supporting medical student wellbeing: lessons from an international research program

Wendy Hu School of Medicine, Western Sydney University, Australia

Eleanor Flynn Melbourne Medical School, University of Melbourne, Australia

Robyn Woodward-Kron Melbourne Medical School, University of Melbourne, Australia

Electronic publication date: 2020 Apr 18

JOURNAL INFORMATION
==============================
NLM Title Abbreviation: Can Med Educ J
Journal ID: Can Med Educ J
NLM Journal ID: 101560935
Journal ID: CMEJ

Canadian Medical Education Journal

EISSN: 1923-1202
Publisher: University of Saskatchewan

ARTICLE INFORMATION
==============================
Subjects: Ob 6–1

TRC in Action: Leading the Way in Increasing the Number of Indigenous Physicians at UBC

James Andrew University of British Columbia

Electronic publication date: 2020 Apr 18

JOURNAL INFORMATION
==============================
NLM Title Abbreviation: Can Med Educ J
Journal ID: Can Med Educ J
NLM Journal ID: 101560935
Journal ID: CMEJ

Canadian Medical Education Journal

EISSN: 1923-1202
Publisher: University of Saskatchewan

ARTICLE INFORMATION
==============================
Subjects: Ob 6–2

The Indigenous KAIROS Blanket Exercise: A Transformative Learning Experience in Medical Education

Lindsay Herzog University of Toronto

Lisa Richardson University of Toronto

Sarah Wright University of Toronto

Jason Pennington University of Toronto

Electronic publication date: 2020 Apr 18

JOURNAL INFORMATION
==============================
NLM Title Abbreviation: Can Med Educ J
Journal ID: Can Med Educ J
NLM Journal ID: 101560935
Journal ID: CMEJ

Canadian Medical Education Journal

EISSN: 1923-1202
Publisher: University of Saskatchewan

ARTICLE INFORMATION
==============================
Subjects: Ob 6–3

Let's talk about the uncomfortable: decolonizing healthcare and educational institutions

Alya Heirali University of Calgary

Rachel Ward University of Calgary

Robert Johnston University of Calgary

Ashley Cornect-Benoit University of Calgary

Sharon Foster University of Calgary

Pearl Yellow Old Woman University of Calgary

Rita Henderson University of Calgary

Cheryl Barnabe University of Calgary

Lynden Crowshoe University of Calgary

Electronic publication date: 2020 Apr 18

JOURNAL INFORMATION
==============================
NLM Title Abbreviation: Can Med Educ J
Journal ID: Can Med Educ J
NLM Journal ID: 101560935
Journal ID: CMEJ

Canadian Medical Education Journal

EISSN: 1923-1202
Publisher: University of Saskatchewan

ARTICLE INFORMATION
==============================
Subjects: Ob 6–4

Creating pathways to healthcare education with Indigenous youth

Natalie Sloof Western University

Layla Amer Ali Western University

Tyler Blue Western University

Brenna Hansen Western University

Danny Kim Western University

George Kim Western University

Electronic publication date: 2020 Apr 18

JOURNAL INFORMATION
==============================
NLM Title Abbreviation: Can Med Educ J
Journal ID: Can Med Educ J
NLM Journal ID: 101560935
Journal ID: CMEJ

Canadian Medical Education Journal

EISSN: 1923-1202
Publisher: University of Saskatchewan

ARTICLE INFORMATION
==============================
Subjects: Ob 6–5

Space for the Sacred in Health Care? The Integration of Indigenous Medicines into Biomedicine

Cathy Fournier Wilson Centre

Robin Oakley Dalhousie University

Lisa Richardson Women's College Hospital

Cynthia Whitehead Wilson Centre

Electronic publication date: 2020 Apr 18

JOURNAL INFORMATION
==============================
NLM Title Abbreviation: Can Med Educ J
Journal ID: Can Med Educ J
NLM Journal ID: 101560935
Journal ID: CMEJ

Canadian Medical Education Journal

EISSN: 1923-1202
Publisher: University of Saskatchewan

ARTICLE INFORMATION
==============================
Subjects: Ob 6–6

Impact of the mini-schools of health on future healthcare professionals' attitudes toward indigenous people

Christophe Moderie McGill

Éric Drouin Université de Montréal

Richard Rioux Université de Montréal

Anne-Sophie Thommeret-Carrière Université de Montréal

Jean-Michel Leduc Université de Montréal

Electronic publication date: 2020 Apr 18

JOURNAL INFORMATION
==============================
NLM Title Abbreviation: Can Med Educ J
Journal ID: Can Med Educ J
NLM Journal ID: 101560935
Journal ID: CMEJ

Canadian Medical Education Journal

EISSN: 1923-1202
Publisher: University of Saskatchewan

ARTICLE INFORMATION
==============================
Subjects: Oc 1–3

The use of Item Parameter Drift and a Multi-Facets Rasch Model to detect potential case exposure in a high-stakes OSCE

Karen Coetzee Touchstone Institute

Sandra Monteiro McMaster University

Electronic publication date: 2020 Apr 18

JOURNAL INFORMATION
==============================
NLM Title Abbreviation: Can Med Educ J
Journal ID: Can Med Educ J
NLM Journal ID: 101560935
Journal ID: CMEJ

Canadian Medical Education Journal

EISSN: 1923-1202
Publisher: University of Saskatchewan

ARTICLE INFORMATION
==============================
Subjects: Oc 1–2

Entrustment and the Objective Structure Clinical Examination (OSCE): bridging the gap towards competency-based medical education

Samantha Halman University of Ottawa

Angel Fu University of Ottawa

Debra Pugh Medical Council of Canada

Electronic publication date: 2020 Apr 18

JOURNAL INFORMATION
==============================
NLM Title Abbreviation: Can Med Educ J
Journal ID: Can Med Educ J
NLM Journal ID: 101560935
Journal ID: CMEJ

Canadian Medical Education Journal

EISSN: 1923-1202
Publisher: University of Saskatchewan

ARTICLE INFORMATION
==============================
Subjects: Oc 1–4

Apples and oranges? Substituting traditional global rating scales with entrustment scales for standard setting in a residency Objective Structured Clinical Exam (OSCE)

Sarah Troster University of Alberta

Vijay Daniels University of Alberta

Deena Hamza University of Alberta

Anna Oswald University of Alberta

Electronic publication date: 2020 Apr 18

JOURNAL INFORMATION
==============================
NLM Title Abbreviation: Can Med Educ J
Journal ID: Can Med Educ J
NLM Journal ID: 101560935
Journal ID: CMEJ

Canadian Medical Education Journal

EISSN: 1923-1202
Publisher: University of Saskatchewan

ARTICLE INFORMATION
==============================
Subjects: Oc 1–5

Factors that influence self-regulation of learning in OSCEs: The role of emotions.

Cynthia Min University of British Columbia

Deborah Butler University of British Columbia

Daniel Pratt University of British Columbia

Kevin Eva University of British Columbia

Electronic publication date: 2020 Apr 18

JOURNAL INFORMATION
==============================
NLM Title Abbreviation: Can Med Educ J
Journal ID: Can Med Educ J
NLM Journal ID: 101560935
Journal ID: CMEJ

Canadian Medical Education Journal

EISSN: 1923-1202
Publisher: University of Saskatchewan

ARTICLE INFORMATION
==============================
Subjects: Oc 1–6

“Why didn't they see my scars?” Critical Discourse Analysis of Simulated Patients' perceived tensions surrounding Objective Structured Clinical Examinations

Helen Reid Queen's University Belfast

Jennifer Johnston Queen's University Belfast

Mairead Corrigan Queen's University Belfast

Tim Dornan Queen's University, Belfast

Electronic publication date: 2020 Apr 18

JOURNAL INFORMATION
==============================
NLM Title Abbreviation: Can Med Educ J
Journal ID: Can Med Educ J
NLM Journal ID: 101560935
Journal ID: CMEJ

Canadian Medical Education Journal

EISSN: 1923-1202
Publisher: University of Saskatchewan

ARTICLE INFORMATION
==============================
Subjects: Oc 2–1

The whole is greater than the sum of its parts: Expanding the breadth of interprofessional education in undergraduate medical education

Nishan Sharma University of Calgary

Rahim Kachra University of Calgary

Cydnee Seneviratne University of Calgary

Electronic publication date: 2020 Apr 18

JOURNAL INFORMATION
==============================
NLM Title Abbreviation: Can Med Educ J
Journal ID: Can Med Educ J
NLM Journal ID: 101560935
Journal ID: CMEJ

Canadian Medical Education Journal

EISSN: 1923-1202
Publisher: University of Saskatchewan

ARTICLE INFORMATION
==============================
Subjects: Oc 2–2

Soutenir le développement des compétences en collaboration interprofessionnelle par la pratique réflexive : une approche innovante

Amélie Richard Université Laval

Emmanuelle Careau Université Laval

Sébastien Yergeau Commission scolaire des Navigateurs

Mathieu Gagnon Université de Sherbrooke

Electronic publication date: 2020 Apr 18

JOURNAL INFORMATION
==============================
NLM Title Abbreviation: Can Med Educ J
Journal ID: Can Med Educ J
NLM Journal ID: 101560935
Journal ID: CMEJ

Canadian Medical Education Journal

EISSN: 1923-1202
Publisher: University of Saskatchewan

ARTICLE INFORMATION
==============================
Subjects: Oc 2–3

Inter-Professional Faculty Development in small groups - the importance of a safe environment.

Erica Amari University of British Columbia

Kiran Veerapen University of British Columbia

Wilson Luong University of British Columbia

Jennifer Clark University of British Columbia

Katherine Wisener University of British Columbia

Brenda Hardie University of British Columbia

Sue Murphy University of British Columbia

Robin Roots University of British Columbia

Donna Drynan University of British Columbia

Julia Klick University of British Columbia

Rose Hatala University of British Columbia

Electronic publication date: 2020 Apr 18

JOURNAL INFORMATION
==============================
NLM Title Abbreviation: Can Med Educ J
Journal ID: Can Med Educ J
NLM Journal ID: 101560935
Journal ID: CMEJ

Canadian Medical Education Journal

EISSN: 1923-1202
Publisher: University of Saskatchewan

ARTICLE INFORMATION
==============================
Subjects: Oc 2–5

What motivates Professional Development? An Interprofessional Glimpse into the Minds of Healthcare Professions Educators

Ingrid Harle Queen’s University

Mikaila De sousa Queen’s University

Eleftherios Soleas Queen’s University

Richard van Wylick Queen’s University

Electronic publication date: 2020 Apr 18

JOURNAL INFORMATION
==============================
NLM Title Abbreviation: Can Med Educ J
Journal ID: Can Med Educ J
NLM Journal ID: 101560935
Journal ID: CMEJ

Canadian Medical Education Journal

EISSN: 1923-1202
Publisher: University of Saskatchewan

ARTICLE INFORMATION
==============================
Subjects: Oc 2–6

Investigating the impact of interprofessional co-debriefing on team performance outcomes in a simulated mass casualty incident.

Irina Charania University of Calgary

Ian Wishart University of Calgary

Gemma Percival University of Calgary

Amy Poelzer University of Calgary

Electronic publication date: 2020 Apr 18

JOURNAL INFORMATION
==============================
NLM Title Abbreviation: Can Med Educ J
Journal ID: Can Med Educ J
NLM Journal ID: 101560935
Journal ID: CMEJ

Canadian Medical Education Journal

EISSN: 1923-1202
Publisher: University of Saskatchewan

ARTICLE INFORMATION
==============================
Subjects: Oc 2–4

“I'll Scratch Your Back If You Scratch Mine…..Maybe.” How helping behaviours are understood and enacted in professional work teams: A Scoping Review.

Erin Kennedy Western University

Sayra Cristancho Western University

Javeed Sukhera Western University

Don Eby Western University

Sandy DeLuca Western University

Electronic publication date: 2020 Apr 18

JOURNAL INFORMATION
==============================
NLM Title Abbreviation: Can Med Educ J
Journal ID: Can Med Educ J
NLM Journal ID: 101560935
Journal ID: CMEJ

Canadian Medical Education Journal

EISSN: 1923-1202
Publisher: University of Saskatchewan

ARTICLE INFORMATION
==============================
Subjects: Oc 3–1

Humanism in Surgery - Developing a Patient as Teacher Initiative in Surgical Clerkship

Jory Simpson University of Toronto

Emilia Kangasjarvi University of Toronto

Allia Karim Reserca

Stella Ng University of Toronto

Electronic publication date: 2020 Apr 18

JOURNAL INFORMATION
==============================
NLM Title Abbreviation: Can Med Educ J
Journal ID: Can Med Educ J
NLM Journal ID: 101560935
Journal ID: CMEJ

Canadian Medical Education Journal

EISSN: 1923-1202
Publisher: University of Saskatchewan

ARTICLE INFORMATION
==============================
Subjects: Oc 3–2

Exploring patient perceptions of learner competence on a medical teaching unit

Megan Mercia University of Calgary

Rahim Kachra University of Calgary

Allison Brown University of Calgary

Irene Ma University of Calgary

Electronic publication date: 2020 Apr 18

JOURNAL INFORMATION
==============================
NLM Title Abbreviation: Can Med Educ J
Journal ID: Can Med Educ J
NLM Journal ID: 101560935
Journal ID: CMEJ

Canadian Medical Education Journal

EISSN: 1923-1202
Publisher: University of Saskatchewan

ARTICLE INFORMATION
==============================
Subjects: Oc 3–3

Patient Involvement in Medical Education Research

Katherine Moreau University of Ottawa

Kaylee Eady University of Ottawa

Sarah Heath University of Ottawa

Electronic publication date: 2020 Apr 18

JOURNAL INFORMATION
==============================
NLM Title Abbreviation: Can Med Educ J
Journal ID: Can Med Educ J
NLM Journal ID: 101560935
Journal ID: CMEJ

Canadian Medical Education Journal

EISSN: 1923-1202
Publisher: University of Saskatchewan

ARTICLE INFORMATION
==============================
Subjects: Oc 3–4

Patient Emancipation? Patient Teacher Programs in Medical Education

Emilia Kangasjarvi University of Toronto

Jory Simpson University of Toronto

Farah Friesen University of Toronto

Stella Ng University of Toronto

Electronic publication date: 2020 Apr 18

JOURNAL INFORMATION
==============================
NLM Title Abbreviation: Can Med Educ J
Journal ID: Can Med Educ J
NLM Journal ID: 101560935
Journal ID: CMEJ

Canadian Medical Education Journal

EISSN: 1923-1202
Publisher: University of Saskatchewan

ARTICLE INFORMATION
==============================
Subjects: Oc 3–5

Fostering non-clinical skills in medical education: a rapid review

Julie Massé Université Laval

Stéphanie Beaura Université Laval

Marie-Claude Tremblay Université Laval

Electronic publication date: 2020 Apr 18

JOURNAL INFORMATION
==============================
NLM Title Abbreviation: Can Med Educ J
Journal ID: Can Med Educ J
NLM Journal ID: 101560935
Journal ID: CMEJ

Canadian Medical Education Journal

EISSN: 1923-1202
Publisher: University of Saskatchewan

ARTICLE INFORMATION
==============================
Subjects: Oc 3–6

How does having the patient continuously present in the teaching and learning encounter affect the learning environment for the attending physician the learner and the patient?

Bavenjit Cheema University of British Columbia

Cheryl Holmes University of British Columbia

Cary Cuncic University of British Columbia

Carolyn Canfield University of British Columbia

Heather Buckley University of British Columbia

Kiran Veerapen University of British Columbia

Erica Amari University of British Columbia

Laura Nimmon University of British Columbia

Daniel Ho University of British Columbia

Anneke van Enk University of British Columbia

Katherine Wisener University of British Columbia

Meredith Li University of British Columbia

Electronic publication date: 2020 Apr 18

JOURNAL INFORMATION
==============================
NLM Title Abbreviation: Can Med Educ J
Journal ID: Can Med Educ J
NLM Journal ID: 101560935
Journal ID: CMEJ

Canadian Medical Education Journal

EISSN: 1923-1202
Publisher: University of Saskatchewan

ARTICLE INFORMATION
==============================
Subjects: Oc 6–1

Social background and medical school choice in the UK: a national qualitative interview study

Eliot Rees University College London

David Harrison University College London

Karen Mattick University of Exeter

Katherine Woolf University College London

Electronic publication date: 2020 Apr 18

JOURNAL INFORMATION
==============================
NLM Title Abbreviation: Can Med Educ J
Journal ID: Can Med Educ J
NLM Journal ID: 101560935
Journal ID: CMEJ

Canadian Medical Education Journal

EISSN: 1923-1202
Publisher: University of Saskatchewan

ARTICLE INFORMATION
==============================
Subjects: Oc 6–6

CASPer Down Under: understanding the role of SJT in selecting rural background applicants for medical school in Australia

Lyndal Parker-Newlyn University of WOLLONGONG

Kylie Mansfield University of Wollongong

Kelly Dore McMaster University

Electronic publication date: 2020 Apr 18

JOURNAL INFORMATION
==============================
NLM Title Abbreviation: Can Med Educ J
Journal ID: Can Med Educ J
NLM Journal ID: 101560935
Journal ID: CMEJ

Canadian Medical Education Journal

EISSN: 1923-1202
Publisher: University of Saskatchewan

ARTICLE INFORMATION
==============================
Subjects: Oc 6–3

ral and Remote Sustainability Score. Eight years of experience as a screening tool for admission to a distributed medical education program (DME) with an emphasis on rural practice.

Tammy Klassen-Ross University of Northern British Columbia

Paul Winwood University of British Columbia

Geoff Payne University of Northern British Columbia

Electronic publication date: 2020 Apr 18

JOURNAL INFORMATION
==============================
NLM Title Abbreviation: Can Med Educ J
Journal ID: Can Med Educ J
NLM Journal ID: 101560935
Journal ID: CMEJ

Canadian Medical Education Journal

EISSN: 1923-1202
Publisher: University of Saskatchewan

ARTICLE INFORMATION
==============================
Subjects: Oc 6–4

Gender and age effect in integrated French Multiple Mini Interviews. A maturity issue?

Robert Gagnon Université de Montréal

Philippe Bégin Université de Montréal

Jean-Michel Leduc Université de Montréal

Christian Bourdy Université de Montréal

Electronic publication date: 2020 Apr 18

JOURNAL INFORMATION
==============================
NLM Title Abbreviation: Can Med Educ J
Journal ID: Can Med Educ J
NLM Journal ID: 101560935
Journal ID: CMEJ

Canadian Medical Education Journal

EISSN: 1923-1202
Publisher: University of Saskatchewan

ARTICLE INFORMATION
==============================
Subjects: Oc 6–5

Nature over Nurture: Selecting students with rural backgrounds is addressing maldistribution of physiotherapists

Robin Roots University of British Columbia

Anne Worthington University of British Columbia

Andrea Gingerich University of Northern British Columbia

Sue Murphy University of British Columbia

Electronic publication date: 2020 Apr 18

JOURNAL INFORMATION
==============================
NLM Title Abbreviation: Can Med Educ J
Journal ID: Can Med Educ J
NLM Journal ID: 101560935
Journal ID: CMEJ

Canadian Medical Education Journal

EISSN: 1923-1202
Publisher: University of Saskatchewan

ARTICLE INFORMATION
==============================
Subjects: Oc 6–2

Associations between CASPer scores and sociodemographic characteristics of applicants : preliminary findings from a multicenter study in Quebec

Jean-Michel Leduc Université de Montréal

Robert Gagnon Université de Montréal

Valeria Akim Université de Montréal

Richard Rioux Université de Montréal

Philippe Bégin Université de Montréal

Natalie Loye Université de Montréal

Sébastien Béland Université de Montréal

Jean-Sébastien Renaud Université Laval

Claire Hudon Université Laval

Martine Bourget Université Laval

Annie Ouellet Université de Sherbrooke

Isabelle Gauthier Université de Sherbrooke

Sondos Samandi Université de Sherbrooke

Estelle Chetrit McGill

Saleem Razack McGill

Kate Hooton McGill

Christian Bourdy Université de Montréal

Electronic publication date: 2020 Apr 18

JOURNAL INFORMATION
==============================
NLM Title Abbreviation: Can Med Educ J
Journal ID: Can Med Educ J
NLM Journal ID: 101560935
Journal ID: CMEJ

Canadian Medical Education Journal

EISSN: 1923-1202
Publisher: University of Saskatchewan

ARTICLE INFORMATION
==============================
Subjects: Oc 4–1

Changing Practitioners' Perspectives: Using first-person perspective in 360-video to cultivate empathy in health professionals

Seema Marwaha University of Toronto

Sara Martel Trillium Health Partners Institute for Better Health

Arjun Sharma University of Toronto

Matthew Strang Trillium Health Partners Institute for Better Health

Nikita Singh Trillium Health Partners Institute for Better Health

Electronic publication date: 2020 Apr 18

JOURNAL INFORMATION
==============================
NLM Title Abbreviation: Can Med Educ J
Journal ID: Can Med Educ J
NLM Journal ID: 101560935
Journal ID: CMEJ

Canadian Medical Education Journal

EISSN: 1923-1202
Publisher: University of Saskatchewan

ARTICLE INFORMATION
==============================
Subjects: Oc 4–2

Health Artificial Intelligence Ethics in Medical Education

Sara Gerke Harvard Law School

Gali Katznelson Western University

Electronic publication date: 2020 Apr 18

JOURNAL INFORMATION
==============================
NLM Title Abbreviation: Can Med Educ J
Journal ID: Can Med Educ J
NLM Journal ID: 101560935
Journal ID: CMEJ

Canadian Medical Education Journal

EISSN: 1923-1202
Publisher: University of Saskatchewan

ARTICLE INFORMATION
==============================
Subjects: Oc 4–3

Creating an “Entrepreneurship in Healthcare” teaching program for medical students at the University of Toronto

Aida Ahrari University of Toronto

Pawandeep Sandhu University of Toronto

Dante Morra University of Toronto

Alison Freeland University of Toronto

Sarah McClennan University of Toronto

Electronic publication date: 2020 Apr 18

JOURNAL INFORMATION
==============================
NLM Title Abbreviation: Can Med Educ J
Journal ID: Can Med Educ J
NLM Journal ID: 101560935
Journal ID: CMEJ

Canadian Medical Education Journal

EISSN: 1923-1202
Publisher: University of Saskatchewan

ARTICLE INFORMATION
==============================
Subjects: Oc 4–4

Digital Compassion: A conceptual framework for learning to care in the digital age

David Wiljer University of Toronto

Rebecca Charow University of Toronto

Gillian Strudwick University of Toronto

Allison Crawford University of Toronto

Electronic publication date: 2020 Apr 18

JOURNAL INFORMATION
==============================
NLM Title Abbreviation: Can Med Educ J
Journal ID: Can Med Educ J
NLM Journal ID: 101560935
Journal ID: CMEJ

Canadian Medical Education Journal

EISSN: 1923-1202
Publisher: University of Saskatchewan

ARTICLE INFORMATION
==============================
Subjects: Oc 4–6

Validation of a grid to document the quality of self-explanation when implemented as a learning strategy at the UGME level

Martine Chamberland Université de Sherbrooke

Jean Setrakian Université de Sherbrooke

Mélanie Marceau Université de Sherbrooke

Élise Vachon Lachiver Université de Sherbrooke

Julie Ouellet Université de Sherbrooke

Émilie Blais Université de Sherbrooke

Linda Bergeron Université de Sherbrooke

Christina St-Onge Université de Sherbrooke

Electronic publication date: 2020 Apr 18

JOURNAL INFORMATION
==============================
NLM Title Abbreviation: Can Med Educ J
Journal ID: Can Med Educ J
NLM Journal ID: 101560935
Journal ID: CMEJ

Canadian Medical Education Journal

EISSN: 1923-1202
Publisher: University of Saskatchewan

ARTICLE INFORMATION
==============================
Subjects: Oc 4–5

Exploring the relationship between participation in project echo and healthcare providers' orientation to lifelong learning

Sanjeev Sockalingam University of Toronto

Thiyake Rajaratnam Centre for Addiction and Mental Health

Amanda Gambin Centre for Addiction and Mental Health

Jenny Hardy Centre for Addiction and Mental Health

Eva Serhal Centre for Addiction and Mental Health

Allison Crawford University of Toronto

Electronic publication date: 2020 Apr 18

JOURNAL INFORMATION
==============================
NLM Title Abbreviation: Can Med Educ J
Journal ID: Can Med Educ J
NLM Journal ID: 101560935
Journal ID: CMEJ

Canadian Medical Education Journal

EISSN: 1923-1202
Publisher: University of Saskatchewan

ARTICLE INFORMATION
==============================
Subjects: Oc 5–1

Curricular Alignment Matrices

Amanda Stalwick University of Saskatchewan

Regina Taylor-Gjevre University of Saskatchewan

Electronic publication date: 2020 Apr 18

JOURNAL INFORMATION
==============================
NLM Title Abbreviation: Can Med Educ J
Journal ID: Can Med Educ J
NLM Journal ID: 101560935
Journal ID: CMEJ

Canadian Medical Education Journal

EISSN: 1923-1202
Publisher: University of Saskatchewan

ARTICLE INFORMATION
==============================
Subjects: Oc 5–2

Using assessment and evaluation data to determine the state of medical programs: A program evaluation framework.

Pauline Pan University of Toronto

David Rojas University of Toronto

Frazer Howard University of Toronto

David Tihanyi University of Toronto

Christopher Jones University of Toronto

Richard Pittini University of Toronto

Electronic publication date: 2020 Apr 18

JOURNAL INFORMATION
==============================
NLM Title Abbreviation: Can Med Educ J
Journal ID: Can Med Educ J
NLM Journal ID: 101560935
Journal ID: CMEJ

Canadian Medical Education Journal

EISSN: 1923-1202
Publisher: University of Saskatchewan

ARTICLE INFORMATION
==============================
Subjects: Oc 5–3

Using design thinking to re-invent the delivery of undergraduate medical education

Rahim Kachra University of Calgary

Allison Brown University of Calgary

Mike Paget University of Calgary

Nishan Sharma University of Calgary

Joshua Low University of Calgary

Kathryne Brockman University of Calgary

Zachary Urquhart University of Calgary

Electronic publication date: 2020 Apr 18

JOURNAL INFORMATION
==============================
NLM Title Abbreviation: Can Med Educ J
Journal ID: Can Med Educ J
NLM Journal ID: 101560935
Journal ID: CMEJ

Canadian Medical Education Journal

EISSN: 1923-1202
Publisher: University of Saskatchewan

ARTICLE INFORMATION
==============================
Subjects: Oc 5–4

Exploring criteria used by judges to rate content for medical education curricula

Marcel D'Eon University of Saskatchewan

Malshi Karunatilake University of Saskatchewan

Greg Malin University of Saskatchewan

Harold Bull University of Saskatchewan

Electronic publication date: 2020 Apr 18

JOURNAL INFORMATION
==============================
NLM Title Abbreviation: Can Med Educ J
Journal ID: Can Med Educ J
NLM Journal ID: 101560935
Journal ID: CMEJ

Canadian Medical Education Journal

EISSN: 1923-1202
Publisher: University of Saskatchewan

ARTICLE INFORMATION
==============================
Subjects: Oc 5–5

Curriculum Renewal as Organizational Learning

Christen Rachul University of Manitoba

Helen Mawdsley University of Manitoba

Benjamin Collins University of Manitoba

Keevin Bernstein University of Manitoba

Ira Ripstein University of Manitoba

Joanne Hamilton University of Manitoba

Electronic publication date: 2020 Apr 18

JOURNAL INFORMATION
==============================
NLM Title Abbreviation: Can Med Educ J
Journal ID: Can Med Educ J
NLM Journal ID: 101560935
Journal ID: CMEJ

Canadian Medical Education Journal

EISSN: 1923-1202
Publisher: University of Saskatchewan

ARTICLE INFORMATION
==============================
Subjects: Oc 5–6

A theory-based stakeholder evaluation of an integrative resilience curriculum

Juehea (Lucia) Lee University of Toronto

Ben Shachar University of Toronto

Joanne Leo University of Toronto

Shayna Kulman-Lipsey University of Toronto

Leslie Nickell University of Toronto

Nellie Perret University of Toronto

David Rojas Gualdron University of Toronto

Electronic publication date: 2020 Apr 18

JOURNAL INFORMATION
==============================
NLM Title Abbreviation: Can Med Educ J
Journal ID: Can Med Educ J
NLM Journal ID: 101560935
Journal ID: CMEJ

Canadian Medical Education Journal

EISSN: 1923-1202
Publisher: University of Saskatchewan

ARTICLE INFORMATION
==============================
Subjects: Oc 7–1

Surgery ABCs: A Healthcare Podcast for Kids

Natalie Marsden University of Alberta

Jenni Marshall University of Alberta

Jonathan White University of Alberta

Electronic publication date: 2020 Apr 18

JOURNAL INFORMATION
==============================
NLM Title Abbreviation: Can Med Educ J
Journal ID: Can Med Educ J
NLM Journal ID: 101560935
Journal ID: CMEJ

Canadian Medical Education Journal

EISSN: 1923-1202
Publisher: University of Saskatchewan

ARTICLE INFORMATION
==============================
Subjects: Oc 7–2

Factors influencing medical student perception of a career in General Surgery

Savannah Silva McMaster University

Ashley Eom McMaster University

Qian Shi McMaster University

Danyal Saeed McMaster University

Jeffery Grab University of Alberta

Patrick Ciechanski University of Alberta

Deepak Dath McMaster University

Electronic publication date: 2020 Apr 18

JOURNAL INFORMATION
==============================
NLM Title Abbreviation: Can Med Educ J
Journal ID: Can Med Educ J
NLM Journal ID: 101560935
Journal ID: CMEJ

Canadian Medical Education Journal

EISSN: 1923-1202
Publisher: University of Saskatchewan

ARTICLE INFORMATION
==============================
Subjects: Oc 7–3

Impact of gaps in surgical training and assessment on performance in an Obstetrics and Gynecology residency program

Stephanie Scott Dalhousie University

Kevin Eva University of British Columbia

Nancy VanEyk Dalhousie University

Electronic publication date: 2020 Apr 18

JOURNAL INFORMATION
==============================
NLM Title Abbreviation: Can Med Educ J
Journal ID: Can Med Educ J
NLM Journal ID: 101560935
Journal ID: CMEJ

Canadian Medical Education Journal

EISSN: 1923-1202
Publisher: University of Saskatchewan

ARTICLE INFORMATION
==============================
Subjects: Oc 7–4

General surgery resident work patterns, results of a time- motion analysis study.

Eric Walser Western University

Chris J Zhang Western University

Sayra Cristancho Western University

Lorelei Lingard Western University

Michael Ott Western University

Anna Mierzwa University of Alberta

Electronic publication date: 2020 Apr 18

JOURNAL INFORMATION
==============================
NLM Title Abbreviation: Can Med Educ J
Journal ID: Can Med Educ J
NLM Journal ID: 101560935
Journal ID: CMEJ

Canadian Medical Education Journal

EISSN: 1923-1202
Publisher: University of Saskatchewan

ARTICLE INFORMATION
==============================
Subjects: Oc 7–5

Propagating the “SEAD”: exploring the value of an overnight call shift in the Surgical Exploration and Discovery Program

Aram Abbasian University of Toronto

Shirley Xue Jiang University of Toronto

James Rutka University of Toronto

Nada Gawad University of Ottawa

Teresa Ziegler University of Toronto

Alexander Adibfar University of Toronto

Electronic publication date: 2020 Apr 18

JOURNAL INFORMATION
==============================
NLM Title Abbreviation: Can Med Educ J
Journal ID: Can Med Educ J
NLM Journal ID: 101560935
Journal ID: CMEJ

Canadian Medical Education Journal

EISSN: 1923-1202
Publisher: University of Saskatchewan

ARTICLE INFORMATION
==============================
Subjects: Oc 7–6

Educational impact drives feasibility of workplace-based assessment for cataract surgery

Nawaaz Nathoo University of British Columbia

Andrea Gingerich University of British Columbia

Ravi Sidhu University of British Columbia

Electronic publication date: 2020 Apr 18

JOURNAL INFORMATION
==============================
NLM Title Abbreviation: Can Med Educ J
Journal ID: Can Med Educ J
NLM Journal ID: 101560935
Journal ID: CMEJ

Canadian Medical Education Journal

EISSN: 1923-1202
Publisher: University of Saskatchewan

ARTICLE INFORMATION
==============================
Subjects: Od 1–1

A Dialogical Approach to Teaching for Person-Centred Care

Victoria Boyd University of Toronto

Lisa Richardson University of Toronto

Paula Veinot Independently employed

Tarek Abdelhalim University of Toronto

Mary Jane Bell University of Toronto

Zac Feilchenfeld University of Toronto

Umberin Najeeb University of Toronto

Dominique Piquette University of Toronto

Shail Rawal University of Toronto

Rene Wong University of Toronto

Sarah Wright University of Toronto

Cynthia Whitehead University of Toronto

Arno Kumagai University of Toronto

Ayelet Kuper University of Toronto

Electronic publication date: 2020 Apr 18

JOURNAL INFORMATION
==============================
NLM Title Abbreviation: Can Med Educ J
Journal ID: Can Med Educ J
NLM Journal ID: 101560935
Journal ID: CMEJ

Canadian Medical Education Journal

EISSN: 1923-1202
Publisher: University of Saskatchewan

ARTICLE INFORMATION
==============================
Subjects: Od 1–2

Perceptions of Incoming Medical Residents about their role as a Teacher

Wilson Luong University of British Columbia

Kiran Veerapen University of British Columbia

Erica Amari University of British Columbia

Jennifer McKay University of British Columbia

Sharon Doucet University of British Columbia

Henry Broekhuyse University of British Columbia

Clarissa Wallace University of British Columbia

Electronic publication date: 2020 Apr 18

JOURNAL INFORMATION
==============================
NLM Title Abbreviation: Can Med Educ J
Journal ID: Can Med Educ J
NLM Journal ID: 101560935
Journal ID: CMEJ

Canadian Medical Education Journal

EISSN: 1923-1202
Publisher: University of Saskatchewan

ARTICLE INFORMATION
==============================
Subjects: Od 1–3

Medical Student POCUS Peer-Teaching: Grassroots Revolution Ready for Mainstream

Claire Heslop University of Toronto

Mazen El-Baba University of Toronto

Katherine Dillon University of Toronto

Kathryn Corbett University of Toronto

Mazen El-Baba University of Toronto

Electronic publication date: 2020 Apr 18

JOURNAL INFORMATION
==============================
NLM Title Abbreviation: Can Med Educ J
Journal ID: Can Med Educ J
NLM Journal ID: 101560935
Journal ID: CMEJ

Canadian Medical Education Journal

EISSN: 1923-1202
Publisher: University of Saskatchewan

ARTICLE INFORMATION
==============================
Subjects: Od 1–4

Teaching medical students political advocacy skills through experiential learning: lessons from the 2019 Canadian Federation of Medical Students (CFMS) Day of Action

Charles Yin Western University

Yipeng Ge University of Ottawa

Electronic publication date: 2020 Apr 18

JOURNAL INFORMATION
==============================
NLM Title Abbreviation: Can Med Educ J
Journal ID: Can Med Educ J
NLM Journal ID: 101560935
Journal ID: CMEJ

Canadian Medical Education Journal

EISSN: 1923-1202
Publisher: University of Saskatchewan

ARTICLE INFORMATION
==============================
Subjects: Od 1–5

The Use of Personal Notes in Medical Practice: Implications for How we Teach Clinical Documentation

Mark Goldszmidt Western University

Lara Varpio F. Edward Hébert School of Medicine

Pamela McKenzie Western University

Electronic publication date: 2020 Apr 18

JOURNAL INFORMATION
==============================
NLM Title Abbreviation: Can Med Educ J
Journal ID: Can Med Educ J
NLM Journal ID: 101560935
Journal ID: CMEJ

Canadian Medical Education Journal

EISSN: 1923-1202
Publisher: University of Saskatchewan

ARTICLE INFORMATION
==============================
Subjects: Od 1–6

Teaching Medicine to a General Public: How to Assess If Your Audience Is Learning

Malgorzata Kaminska University Of Northern British Columbia

Trina Fyfe University Of Northern British Columbia

Cirisse Stephen University of British Columbia

Lisa Munro University Of Northern British Columbia

Sonya Kruger University Of Northern British Columbia

Lindsay Mathews University Of Northern British Columbia

Peyton Fisher University of British Columbia

Electronic publication date: 2020 Apr 18

JOURNAL INFORMATION
==============================
NLM Title Abbreviation: Can Med Educ J
Journal ID: Can Med Educ J
NLM Journal ID: 101560935
Journal ID: CMEJ

Canadian Medical Education Journal

EISSN: 1923-1202
Publisher: University of Saskatchewan

ARTICLE INFORMATION
==============================
Subjects: Od 2–1

Increasing instructional relevance based on student reflections after they search for evidence

Maria C Tan University of Alberta

Sandy Campbell University of Alberta

Electronic publication date: 2020 Apr 18

JOURNAL INFORMATION
==============================
NLM Title Abbreviation: Can Med Educ J
Journal ID: Can Med Educ J
NLM Journal ID: 101560935
Journal ID: CMEJ

Canadian Medical Education Journal

EISSN: 1923-1202
Publisher: University of Saskatchewan

ARTICLE INFORMATION
==============================
Subjects: Od 2–2

Experiential learning in clinical research: Lessons learnt from a transplant educational training model.

Olusegun Famure University of Toronto

Bronte Anderson University of Toronto

Angel Cai McMaster University

Joseph Kim University of Toronto

Electronic publication date: 2020 Apr 18

JOURNAL INFORMATION
==============================
NLM Title Abbreviation: Can Med Educ J
Journal ID: Can Med Educ J
NLM Journal ID: 101560935
Journal ID: CMEJ

Canadian Medical Education Journal

EISSN: 1923-1202
Publisher: University of Saskatchewan

ARTICLE INFORMATION
==============================
Subjects: Od 2–3

The Best Practices In Medicine (BPiM) Project - Exploring the Use of Intitutional Data and E-learning Modules to Improve Resource Utilization

Rishie Seth University of Toronto

Elizabeth Wooster University of Toronto

Mallory Jackman University of Toronto

Jerry Maniate University of Ottawa

Electronic publication date: 2020 Apr 18

JOURNAL INFORMATION
==============================
NLM Title Abbreviation: Can Med Educ J
Journal ID: Can Med Educ J
NLM Journal ID: 101560935
Journal ID: CMEJ

Canadian Medical Education Journal

EISSN: 1923-1202
Publisher: University of Saskatchewan

ARTICLE INFORMATION
==============================
Subjects: Od 2–4

Using experiential learning to train physicians and teams in obesity prevention and management: 5As Team Multidisciplinary Training Program.

Denise Campbell-Scherer University of Alberta

Melanie Heatherington University of Alberta

Dayna Lee-Baggley Dalhousie University

Thea Luig University of Alberta

Guillermina Noël Luzern University of Applied Sciences & Arts

Sonja Wicklum University of Calgary

Angie Hong University of Toronto

Erin Cameron Northern Ontario School of Medicine

Electronic publication date: 2020 Apr 18

JOURNAL INFORMATION
==============================
NLM Title Abbreviation: Can Med Educ J
Journal ID: Can Med Educ J
NLM Journal ID: 101560935
Journal ID: CMEJ

Canadian Medical Education Journal

EISSN: 1923-1202
Publisher: University of Saskatchewan

ARTICLE INFORMATION
==============================
Subjects: Od 2–5

Exploring the Role of Social Networks in Supporting Faculty Development

Heather Buckley University of British Columbia

Laura Nimmon University of British Columbia

Electronic publication date: 2020 Apr 18

JOURNAL INFORMATION
==============================
NLM Title Abbreviation: Can Med Educ J
Journal ID: Can Med Educ J
NLM Journal ID: 101560935
Journal ID: CMEJ

Canadian Medical Education Journal

EISSN: 1923-1202
Publisher: University of Saskatchewan

ARTICLE INFORMATION
==============================
Subjects: Od 2–6

Material Concepts: A Randomized Trial Exploring Simulation as a Medium to Enhance Cognitive Integration and Transfer of Learning

Kulamakan Kulasegaram University of Toronto

Nicole Woods University of Toronto

Ryan Brydges University of Toronto

Jeffrey Cheung University of Illinois at Chicago College of Medicine

Electronic publication date: 2020 Apr 18

JOURNAL INFORMATION
==============================
NLM Title Abbreviation: Can Med Educ J
Journal ID: Can Med Educ J
NLM Journal ID: 101560935
Journal ID: CMEJ

Canadian Medical Education Journal

EISSN: 1923-1202
Publisher: University of Saskatchewan

ARTICLE INFORMATION
==============================
Subjects: Od 6–1

Multi-source Feedback: Components Supporting Quality Improvement

Marguerite Roy Medical Council of Canada

Claire Touchie Medical Council of Canada

Nichole Kain College of Physicians and Surgeons of Alberta

Electronic publication date: 2020 Apr 18

JOURNAL INFORMATION
==============================
NLM Title Abbreviation: Can Med Educ J
Journal ID: Can Med Educ J
NLM Journal ID: 101560935
Journal ID: CMEJ

Canadian Medical Education Journal

EISSN: 1923-1202
Publisher: University of Saskatchewan

ARTICLE INFORMATION
==============================
Subjects: Od 6–2

Development and Evaluation of a Multisource Feedback (MSF) Form for Residency Program Director Competencies

Theresa Beesley McGill

Evelyn Constantin McGill

Mara Kontopoulos McGill

Patricia Wade McGill

Armand Aalamian McGill

Electronic publication date: 2020 Apr 18

JOURNAL INFORMATION
==============================
NLM Title Abbreviation: Can Med Educ J
Journal ID: Can Med Educ J
NLM Journal ID: 101560935
Journal ID: CMEJ

Canadian Medical Education Journal

EISSN: 1923-1202
Publisher: University of Saskatchewan

ARTICLE INFORMATION
==============================
Subjects: Od 6–3

Perceptions of Assessment and Feedback: Relationships and Reconciliation

Kaif Pardhan University of Toronto

Christopher Watling Western University

Linda Jones University of Dundee

Electronic publication date: 2020 Apr 18

JOURNAL INFORMATION
==============================
NLM Title Abbreviation: Can Med Educ J
Journal ID: Can Med Educ J
NLM Journal ID: 101560935
Journal ID: CMEJ

Canadian Medical Education Journal

EISSN: 1923-1202
Publisher: University of Saskatchewan

ARTICLE INFORMATION
==============================
Subjects: Od 6–4

Multi-source feedback during simulated resuscitation scenarios: a qualitative analysis

Brent Thoma University of Saskatchewan

Tim Chaplin Queen’s University

Adam Szulewski Queen’s University

Heather Braund Queen’s University

Nancy Dalgarno Queen’s University

Rylan Egan Queen’s University

Electronic publication date: 2020 Apr 18

JOURNAL INFORMATION
==============================
NLM Title Abbreviation: Can Med Educ J
Journal ID: Can Med Educ J
NLM Journal ID: 101560935
Journal ID: CMEJ

Canadian Medical Education Journal

EISSN: 1923-1202
Publisher: University of Saskatchewan

ARTICLE INFORMATION
==============================
Subjects: Od 6–5

Multisource feedback of interprofessional competencies: how communication and collaboration are viewed across health disciplines

Kerry Wilbur University of British Columbia

Erik Driessen Maastricht University

Fedde Scheele VU School of Medical Sciences

Pim Teunissen VU Medical Center

Electronic publication date: 2020 Apr 18

JOURNAL INFORMATION
==============================
NLM Title Abbreviation: Can Med Educ J
Journal ID: Can Med Educ J
NLM Journal ID: 101560935
Journal ID: CMEJ

Canadian Medical Education Journal

EISSN: 1923-1202
Publisher: University of Saskatchewan

ARTICLE INFORMATION
==============================
Subjects: Od 6–6

Physician reactions to multisource feedback data and facilitated feedback

Claire Touchie Medical Council of Canada

Marguerite Roy Medical Council of Canada

Nicole Kain College of Physicians and Surgeons of Alberta

Electronic publication date: 2020 Apr 18

JOURNAL INFORMATION
==============================
NLM Title Abbreviation: Can Med Educ J
Journal ID: Can Med Educ J
NLM Journal ID: 101560935
Journal ID: CMEJ

Canadian Medical Education Journal

EISSN: 1923-1202
Publisher: University of Saskatchewan

ARTICLE INFORMATION
==============================
Subjects: Od 7–1

Identity Work in Medical Schools: The Case of Academic Family Physicians

Charo Rodriguez McGill

Emmanuelle Belanger Brown University

Laure Fiquet Universite de Rennes 1

Teresa Pawlikowska Royal College of Surgeons in Ireland

Sofia Lopez-Roig Universidad Miguel Hernandez

Maria Angeles Pastor-Mira Universidad Miguel Hernandez

François-Xavier Schweyer Ecoles des Hauts Etudes en Sante Publique de Rennes

Salvador Pertusa Centro de salud Cabo Huertas

Pierre-Paul Tellier McGill

Electronic publication date: 2020 Apr 18

JOURNAL INFORMATION
==============================
NLM Title Abbreviation: Can Med Educ J
Journal ID: Can Med Educ J
NLM Journal ID: 101560935
Journal ID: CMEJ

Canadian Medical Education Journal

EISSN: 1923-1202
Publisher: University of Saskatchewan

ARTICLE INFORMATION
==============================
Subjects: Od 7–2

Variables associated with students' a priori discipline choices at the time of entry into medical school

Melinda Davis University of Calgary

Janeve Desy University of Calgary

Sue-Ann Facchini University of Calgary

Mike Paget University of Calgary

Sylvain Coderre University of Calgary

Christopher Naugler University of Calgary

Kevin McLaughlin University of Calgary

Electronic publication date: 2020 Apr 18

JOURNAL INFORMATION
==============================
NLM Title Abbreviation: Can Med Educ J
Journal ID: Can Med Educ J
NLM Journal ID: 101560935
Journal ID: CMEJ

Canadian Medical Education Journal

EISSN: 1923-1202
Publisher: University of Saskatchewan

ARTICLE INFORMATION
==============================
Subjects: Od 7–3

Embedding identity: The sense-making process of teachers in family medicine to reconcile their multiple professional identities

David Ortiz-Paredes McGill

Charo Rodriguez McGill

Torsten Risør The Arctic University of Norway

Tamara Carver McGill

Peter Nugus McGill

Electronic publication date: 2020 Apr 18

JOURNAL INFORMATION
==============================
NLM Title Abbreviation: Can Med Educ J
Journal ID: Can Med Educ J
NLM Journal ID: 101560935
Journal ID: CMEJ

Canadian Medical Education Journal

EISSN: 1923-1202
Publisher: University of Saskatchewan

ARTICLE INFORMATION
==============================
Subjects: Od 7–6

La formation de l'identité professionnelle d'enseignant par un programme de formation en pédagogie dans un campus décentralisé

Marie-Hélène Girouard Université de Montréal

Diane Robert Université de Montréal

Caroline Bell Université de Montréal

Julie Morisset Université de Montréal

Electronic publication date: 2020 Apr 18

JOURNAL INFORMATION
==============================
NLM Title Abbreviation: Can Med Educ J
Journal ID: Can Med Educ J
NLM Journal ID: 101560935
Journal ID: CMEJ

Canadian Medical Education Journal

EISSN: 1923-1202
Publisher: University of Saskatchewan

ARTICLE INFORMATION
==============================
Subjects: Od 7–4

Unpacking the Very Visible Knapsack: A Socio-material Analysis of #myCMAbackpack

Alice Cavanagh McMaster University

Electronic publication date: 2020 Apr 18

JOURNAL INFORMATION
==============================
NLM Title Abbreviation: Can Med Educ J
Journal ID: Can Med Educ J
NLM Journal ID: 101560935
Journal ID: CMEJ

Canadian Medical Education Journal

EISSN: 1923-1202
Publisher: University of Saskatchewan

ARTICLE INFORMATION
==============================
Subjects: Od 7–5

How medical students make meaning of early significant clinical experiences: The role of social networks

Samantha Stasiuk University of British Columbia

Laura Nimmon University of British Columbia

Maria Hubinette University of British Columbia

Electronic publication date: 2020 Apr 18

JOURNAL INFORMATION
==============================
NLM Title Abbreviation: Can Med Educ J
Journal ID: Can Med Educ J
NLM Journal ID: 101560935
Journal ID: CMEJ

Canadian Medical Education Journal

EISSN: 1923-1202
Publisher: University of Saskatchewan

ARTICLE INFORMATION
==============================
Subjects: Od 4–1

An online CPD program on keeping medical records using a serious gaming approach. You can't be serious, right?

Martin Tremblay Fédération des médecins spécialistes du Québec

Sam Daniel Fédération des médecins spécialistes du Québec

Beatriz Merlos Fédération des médecins spécialistes du Québec

Electronic publication date: 2020 Apr 18

JOURNAL INFORMATION
==============================
NLM Title Abbreviation: Can Med Educ J
Journal ID: Can Med Educ J
NLM Journal ID: 101560935
Journal ID: CMEJ

Canadian Medical Education Journal

EISSN: 1923-1202
Publisher: University of Saskatchewan

ARTICLE INFORMATION
==============================
Subjects: Od 4–2

Practice changes following participation in a multimodal “CPD-Reality” training: a 6-month follow-up qualitative study

Émilie Gosselin Université de Sherbrooke

Catherine Bertholet Université de Sherbrooke

Marie-France Langlois Université de Sherbrooke

Annie Ouellet Université de Sherbrooke

Electronic publication date: 2020 Apr 18

JOURNAL INFORMATION
==============================
NLM Title Abbreviation: Can Med Educ J
Journal ID: Can Med Educ J
NLM Journal ID: 101560935
Journal ID: CMEJ

Canadian Medical Education Journal

EISSN: 1923-1202
Publisher: University of Saskatchewan

ARTICLE INFORMATION
==============================
Subjects: Od 4–3

Management of Patients with Morbid Obesity in Primary Care: Informing a CPD Event

Nancy Dalgarno Queen’s University

Boris Zevin Queen’s University

Mary Martin Queen’s University

Colleen Grady Queen’s University

Karen Smith Queen’s University

Rachael Morkem Queen’s University

Robyn Houlden Queen’s University

Richard Birtwhistle Queen’s University

David Barber Queen’s University

Electronic publication date: 2020 Apr 18

JOURNAL INFORMATION
==============================
NLM Title Abbreviation: Can Med Educ J
Journal ID: Can Med Educ J
NLM Journal ID: 101560935
Journal ID: CMEJ

Canadian Medical Education Journal

EISSN: 1923-1202
Publisher: University of Saskatchewan

ARTICLE INFORMATION
==============================
Subjects: Od 4–4

Who are you? The roles of practice remediators

Glenn Regehr University of British Columbia

Pim Teunissen School of Health Professions Education

Lara Varpio Uniformed Services University of the Health Sciences

Gisele Bourgeois-Law University of British Columbia

Electronic publication date: 2020 Apr 18

JOURNAL INFORMATION
==============================
NLM Title Abbreviation: Can Med Educ J
Journal ID: Can Med Educ J
NLM Journal ID: 101560935
Journal ID: CMEJ

Canadian Medical Education Journal

EISSN: 1923-1202
Publisher: University of Saskatchewan

ARTICLE INFORMATION
==============================
Subjects: Od 4–5

Primary Care Providers Participation in Cardiology-related Continuing Medical Education Increases the Likelihood of Prescribing Recommended Lipid Management

Diana Sanchez-Ramirez University of Manitoba

Alexander Singer University of Manitoba

Leanne Kosowan University of Manitoba

Alan Katz University of Manitoba

Christine Polimeni University of Manitoba

Electronic publication date: 2020 Apr 18

JOURNAL INFORMATION
==============================
NLM Title Abbreviation: Can Med Educ J
Journal ID: Can Med Educ J
NLM Journal ID: 101560935
Journal ID: CMEJ

Canadian Medical Education Journal

EISSN: 1923-1202
Publisher: University of Saskatchewan

ARTICLE INFORMATION
==============================
Subjects: Od 4–6

A Competency-Based Continuing Professional Development for Routine Pessary Care in Primary Care

Parisa Rezaiefar University of Ottawa

Claire Kendal University of Ottawa

Janine Schieck McKay Montfort hospital

Douglas Archibald University of Ottawa

Maddie Venables Bruyere Research Institute

Electronic publication date: 2020 Apr 18

JOURNAL INFORMATION
==============================
NLM Title Abbreviation: Can Med Educ J
Journal ID: Can Med Educ J
NLM Journal ID: 101560935
Journal ID: CMEJ

Canadian Medical Education Journal

EISSN: 1923-1202
Publisher: University of Saskatchewan

ARTICLE INFORMATION
==============================
Subjects: Od 3–1

The Team is the Point: Structure and Expertise in an Accreditation and Quality Improvement Unit

Patricia Wade McGill

Fernanda Claudio McGill

Electronic publication date: 2020 Apr 18

JOURNAL INFORMATION
==============================
NLM Title Abbreviation: Can Med Educ J
Journal ID: Can Med Educ J
NLM Journal ID: 101560935
Journal ID: CMEJ

Canadian Medical Education Journal

EISSN: 1923-1202
Publisher: University of Saskatchewan

ARTICLE INFORMATION
==============================
Subjects: Od 3–2

Program and Session Evaluations: Leveraging Response Rates

Clare Cook Northern Ontario School of Medicine

James Goertzen Northern Ontario School of Medicine

Electronic publication date: 2020 Apr 18

JOURNAL INFORMATION
==============================
NLM Title Abbreviation: Can Med Educ J
Journal ID: Can Med Educ J
NLM Journal ID: 101560935
Journal ID: CMEJ

Canadian Medical Education Journal

EISSN: 1923-1202
Publisher: University of Saskatchewan

ARTICLE INFORMATION
==============================
Subjects: Od 3–3

Using Regulatory Data to Shift the Performance Curve: MD Snapshot Reports

Nicole Kain College of Physicians & Surgeons of Alberta

Nigel Ashworth University of Alberta

Delaney Wiebe College of Physicians & Surgeons of Alberta

Ed Jess College of Physicians & Surgeons of Alberta

Jacqueline Wagner College of Physicians & Surgeons of Alberta

Nancy Hernandez-Ceron College of Physicians & Surgeons of Alberta

Electronic publication date: 2020 Apr 18

JOURNAL INFORMATION
==============================
NLM Title Abbreviation: Can Med Educ J
Journal ID: Can Med Educ J
NLM Journal ID: 101560935
Journal ID: CMEJ

Canadian Medical Education Journal

EISSN: 1923-1202
Publisher: University of Saskatchewan

ARTICLE INFORMATION
==============================
Subjects: Od 3–4

Learner driven faculty development

James Goertzen Northern Ontario School of Medicine

Clare Cook Northern Ontario School of Medicine

Electronic publication date: 2020 Apr 18

JOURNAL INFORMATION
==============================
NLM Title Abbreviation: Can Med Educ J
Journal ID: Can Med Educ J
NLM Journal ID: 101560935
Journal ID: CMEJ

Canadian Medical Education Journal

EISSN: 1923-1202
Publisher: University of Saskatchewan

ARTICLE INFORMATION
==============================
Subjects: Od 3–5

Exploring how best to teach quality improvement throughout the continuum of medical training: a realist review of peer-reviewed and grey literature.

Allison Brown University of Calgary

Kyle Lafreniere University of Calgary

David Freedman University of Toronto

Aditya Nidumolu Dalhousie University

Matthew Mancuso University of Alberta

Kent Hecker University of Calgary

Aliya Kassam University of Calgary

Electronic publication date: 2020 Apr 18

JOURNAL INFORMATION
==============================
NLM Title Abbreviation: Can Med Educ J
Journal ID: Can Med Educ J
NLM Journal ID: 101560935
Journal ID: CMEJ

Canadian Medical Education Journal

EISSN: 1923-1202
Publisher: University of Saskatchewan

ARTICLE INFORMATION
==============================
Subjects: Od 3–6

Moving away from the “check box” metaphor: how residents are learning about quality improvement during residency training

Aliya Kassam University of Calgary

Allison Brown University of Calgary

Kayla Atchison University of Calgary

Kent Hecker University of Calgary

Electronic publication date: 2020 Apr 18

JOURNAL INFORMATION
==============================
NLM Title Abbreviation: Can Med Educ J
Journal ID: Can Med Educ J
NLM Journal ID: 101560935
Journal ID: CMEJ

Canadian Medical Education Journal

EISSN: 1923-1202
Publisher: University of Saskatchewan

ARTICLE INFORMATION
==============================
Subjects: Od 5–1

Exploring the Effects of Medical Student Absenteeism

Bochra Kurabi University of Toronto

Pauline Pan University of Toronto

Hana Lee University of Toronto

Electronic publication date: 2020 Apr 18

JOURNAL INFORMATION
==============================
NLM Title Abbreviation: Can Med Educ J
Journal ID: Can Med Educ J
NLM Journal ID: 101560935
Journal ID: CMEJ

Canadian Medical Education Journal

EISSN: 1923-1202
Publisher: University of Saskatchewan

ARTICLE INFORMATION
==============================
Subjects: Od 5–2

The Canadian Medical Student Ultrasound Curriculum: Providing Common Ground Where None Exists. A Statement from the Canadian Ultrasound Consensus for Undergraduate Medical Education (CanUCMe) Group

Stephen Miller Dalhousie University

Irene Ma University of Calgary

Peter Steinmetz McGill

Electronic publication date: 2020 Apr 18

JOURNAL INFORMATION
==============================
NLM Title Abbreviation: Can Med Educ J
Journal ID: Can Med Educ J
NLM Journal ID: 101560935
Journal ID: CMEJ

Canadian Medical Education Journal

EISSN: 1923-1202
Publisher: University of Saskatchewan

ARTICLE INFORMATION
==============================
Subjects: Od 5–3

Entering Medical Students' Transition to PBL - an Enhanced Curriculum

Jennifer MacKenzie McMaster University

Lori-Ann Linkins McMaster University

Karen McAssey McMaster University

Robert Whyte McMaster University

Electronic publication date: 2020 Apr 18

JOURNAL INFORMATION
==============================
NLM Title Abbreviation: Can Med Educ J
Journal ID: Can Med Educ J
NLM Journal ID: 101560935
Journal ID: CMEJ

Canadian Medical Education Journal

EISSN: 1923-1202
Publisher: University of Saskatchewan

ARTICLE INFORMATION
==============================
Subjects: Od 5–4

“Am I Meeting That Standard?” -- How Medical Students Set Standards Amid Ill-Defined Expectations

Kevin Chien University of Ottawa

Lorenzo Madrazo Western University

Kori LaDonna University of Ottawa

Electronic publication date: 2020 Apr 18

JOURNAL INFORMATION
==============================
NLM Title Abbreviation: Can Med Educ J
Journal ID: Can Med Educ J
NLM Journal ID: 101560935
Journal ID: CMEJ

Canadian Medical Education Journal

EISSN: 1923-1202
Publisher: University of Saskatchewan

ARTICLE INFORMATION
==============================
Subjects: Od 5–5

Examining the evolution of student perceptions of reflective portfolio in undergraduate medical education

David Li University of British Columbia

Ian Miao Northern Ontario School of Medicine

Maria Hubinette University of British Columbia

Electronic publication date: 2020 Apr 18

JOURNAL INFORMATION
==============================
NLM Title Abbreviation: Can Med Educ J
Journal ID: Can Med Educ J
NLM Journal ID: 101560935
Journal ID: CMEJ

Canadian Medical Education Journal

EISSN: 1923-1202
Publisher: University of Saskatchewan

ARTICLE INFORMATION
==============================
Subjects: Od 5–6

Transforming a Library Resources Lecture to a Show Production for Medical Students: 3 years in the making

Anthony Seto University of Calgary

Zahra Premji University of Calgary

Electronic publication date: 2020 Apr 18

JOURNAL INFORMATION
==============================
NLM Title Abbreviation: Can Med Educ J
Journal ID: Can Med Educ J
NLM Journal ID: 101560935
Journal ID: CMEJ

Canadian Medical Education Journal

EISSN: 1923-1202
Publisher: University of Saskatchewan

ARTICLE INFORMATION
==============================
Subjects: Oe 1–1

Community Physician Retention in South Western Ontario: Perceptions of Longstanding and Recently-Recruited Physicians

Eric Liu Western University

Alexandra Rocha Western University

Robert Mcallister Western University

Danny Kim Western University

George Kim Western University

Electronic publication date: 2020 Apr 18

JOURNAL INFORMATION
==============================
NLM Title Abbreviation: Can Med Educ J
Journal ID: Can Med Educ J
NLM Journal ID: 101560935
Journal ID: CMEJ

Canadian Medical Education Journal

EISSN: 1923-1202
Publisher: University of Saskatchewan

ARTICLE INFORMATION
==============================
Subjects: Oe 1–2

Causes of prescribing errors in children in relation to doctors' prescribing behaviour. Qualitative evaluation using reported incidents

Richard Conn Queen's University Belfast

Mary Tully University of Manchester

Mike Shields Queen's University Belfast

Tim Dornan Queen's University Belfast

Electronic publication date: 2020 Apr 18

JOURNAL INFORMATION
==============================
NLM Title Abbreviation: Can Med Educ J
Journal ID: Can Med Educ J
NLM Journal ID: 101560935
Journal ID: CMEJ

Canadian Medical Education Journal

EISSN: 1923-1202
Publisher: University of Saskatchewan

ARTICLE INFORMATION
==============================
Subjects: Oe 1–3

Examining the unintended effects of intraprofessional (primary-specialty) care models using a critical social theoretical lens

Rene Wong University of Toronto

Simon Kitto University of Ottawa

Cynthia Whitehead University of Toronto

Electronic publication date: 2020 Apr 18

JOURNAL INFORMATION
==============================
NLM Title Abbreviation: Can Med Educ J
Journal ID: Can Med Educ J
NLM Journal ID: 101560935
Journal ID: CMEJ

Canadian Medical Education Journal

EISSN: 1923-1202
Publisher: University of Saskatchewan

ARTICLE INFORMATION
==============================
Subjects: Oe 1–4

Representations of Administrative Staff and Faculty over 50 Years of 'Reports from the Dean'

Morag Paton University of Toronto

Stephanie Waterman University of Toronto

Cynthia Whitehead University of Toronto

Ayelet Kuper University of Toronto

Electronic publication date: 2020 Apr 18

JOURNAL INFORMATION
==============================
NLM Title Abbreviation: Can Med Educ J
Journal ID: Can Med Educ J
NLM Journal ID: 101560935
Journal ID: CMEJ

Canadian Medical Education Journal

EISSN: 1923-1202
Publisher: University of Saskatchewan

ARTICLE INFORMATION
==============================
Subjects: Oe 1–5

Perceptions of Research by Schulich School of Medicine & Dentistry Physicians Located in an Academic Centre or Distributed Sites

Lynn Doan Western University

Madeline Taylor Western University

Tommaso Romagnoli Western University

Danny Kim Western University

George Kim Western University

Electronic publication date: 2020 Apr 18

JOURNAL INFORMATION
==============================
NLM Title Abbreviation: Can Med Educ J
Journal ID: Can Med Educ J
NLM Journal ID: 101560935
Journal ID: CMEJ

Canadian Medical Education Journal

EISSN: 1923-1202
Publisher: University of Saskatchewan

ARTICLE INFORMATION
==============================
Subjects: Oe 1–6

How the Certificates of Added Competence shapes family medicine practice in Canada

Meredith Vanstone McMaster University

Ilana Allice McMaster University

Alison Baker McMaster University

Alexandra Farag McMaster University

Jesse Guscott McMaster University

Michelle Howard McMaster University

Margo Mountjoy McMaster University

Henry Siu McMaster University

X. Catherine Tong McMaster University

Lawrence Grierson McMaster University

Electronic publication date: 2020 Apr 18

JOURNAL INFORMATION
==============================
NLM Title Abbreviation: Can Med Educ J
Journal ID: Can Med Educ J
NLM Journal ID: 101560935
Journal ID: CMEJ

Canadian Medical Education Journal

EISSN: 1923-1202
Publisher: University of Saskatchewan

ARTICLE INFORMATION
==============================
Subjects: Oe 2–1

Structured Oral Exams: Exploring Stakeholders' Perspectives

Isabelle Boulais Université de Sherbrooke

Frédéric Bernier Université de Sherbrooke

Elise Vachon Lachiver Université de Sherbrooke

Melanie Marceau Université de Sherbrooke

Linda Bergeron Université de Sherbrooke

Christina St-Onge Université de Sherbrooke

Electronic publication date: 2020 Apr 18

JOURNAL INFORMATION
==============================
NLM Title Abbreviation: Can Med Educ J
Journal ID: Can Med Educ J
NLM Journal ID: 101560935
Journal ID: CMEJ

Canadian Medical Education Journal

EISSN: 1923-1202
Publisher: University of Saskatchewan

ARTICLE INFORMATION
==============================
Subjects: Oe 2–2

Evaluation of Entrustable Professional Activities Assessment in Undergraduate Medical Education using Mobile Technology

Vernon Curran Memorial University of Newfoundland

Nicholas Fairbridge Memorial University of Newfoundland

Diana Deacon Memorial University of Newfoundland

Norah Duggan Memorial University of Newfoundland

Katherine Stringer Memorial University of Newfoundland

Heidi Coombs Memorial University of Newfoundland

Steve Pennell Memorial University of Newfoundland

Electronic publication date: 2020 Apr 18

JOURNAL INFORMATION
==============================
NLM Title Abbreviation: Can Med Educ J
Journal ID: Can Med Educ J
NLM Journal ID: 101560935
Journal ID: CMEJ

Canadian Medical Education Journal

EISSN: 1923-1202
Publisher: University of Saskatchewan

ARTICLE INFORMATION
==============================
Subjects: Oe 2–3

Learner Handover - Who is it really for?

Susan Humphrey-Murto University of Ottawa

Lorelei Lingard Western University

Lara Varpio Uniformed Services University of the Health Sciences

Chris Watling Western University

Shiphra Ginsburg University of Toronto

Kori LaDonna University of Ottawa

Electronic publication date: 2020 Apr 18

JOURNAL INFORMATION
==============================
NLM Title Abbreviation: Can Med Educ J
Journal ID: Can Med Educ J
NLM Journal ID: 101560935
Journal ID: CMEJ

Canadian Medical Education Journal

EISSN: 1923-1202
Publisher: University of Saskatchewan

ARTICLE INFORMATION
==============================
Subjects: Oe 2–4

Workplace medical student mistreatment: assessment of interventions

Maury Pinsk University of Manitoba

Gillian Nattress University of Manitoba

Electronic publication date: 2020 Apr 18

JOURNAL INFORMATION
==============================
NLM Title Abbreviation: Can Med Educ J
Journal ID: Can Med Educ J
NLM Journal ID: 101560935
Journal ID: CMEJ

Canadian Medical Education Journal

EISSN: 1923-1202
Publisher: University of Saskatchewan

ARTICLE INFORMATION
==============================
Subjects: Oe 2–5

Seeing but not believing: Insights into the intractability of failure to fail

Andrea Gingerich University of British Columbia

Stefanie Sebok-Syer Stanford University

Roseann Larstone University of Northern British Columbia

Christopher Watling Western University

Lorelei Lingard Western University

Electronic publication date: 2020 Apr 18

JOURNAL INFORMATION
==============================
NLM Title Abbreviation: Can Med Educ J
Journal ID: Can Med Educ J
NLM Journal ID: 101560935
Journal ID: CMEJ

Canadian Medical Education Journal

EISSN: 1923-1202
Publisher: University of Saskatchewan

ARTICLE INFORMATION
==============================
Subjects: Oe 2–6

Idiosyncrasy in assessment comments: Do faculty have distinct writing styles?

Andrea Gingerich University of British Columbia

Christopher Watling Western University

Kevin Eva University of British Columbia

Shiphra Ginsburg University of Toronto

Electronic publication date: 2020 Apr 18

JOURNAL INFORMATION
==============================
NLM Title Abbreviation: Can Med Educ J
Journal ID: Can Med Educ J
NLM Journal ID: 101560935
Journal ID: CMEJ

Canadian Medical Education Journal

EISSN: 1923-1202
Publisher: University of Saskatchewan

ARTICLE INFORMATION
==============================
Subjects: Oe 3–1

Creation of a Pilot Addiction Medicine Week Created for and by Pre-Clerkship Medical Students

Melissa Tigert University of Toronto

Hilary Stone University of Toronto

Ruby Alvi University of Toronto

Peter Selby University of Toronto

Azadeh Moaveni University of Toronto

Joyce Nyhof-Young University of Toronto

Robin Glicksman University of Toronto

Electronic publication date: 2020 Apr 18

JOURNAL INFORMATION
==============================
NLM Title Abbreviation: Can Med Educ J
Journal ID: Can Med Educ J
NLM Journal ID: 101560935
Journal ID: CMEJ

Canadian Medical Education Journal

EISSN: 1923-1202
Publisher: University of Saskatchewan

ARTICLE INFORMATION
==============================
Subjects: Oe 3–2

Who Wants to Translate? Evaluation of a Novel Medical Mandarin Education Program for and by Pre-clerkship Medical Students

Jin Sheng (Jason) Zhou University of Toronto

RuiQi Chen University of Toronto

Yu Yang Feng University of Toronto

Yao Lu University of Toronto

Joyce Nyhof-Young University of Toronto

Electronic publication date: 2020 Apr 18

JOURNAL INFORMATION
==============================
NLM Title Abbreviation: Can Med Educ J
Journal ID: Can Med Educ J
NLM Journal ID: 101560935
Journal ID: CMEJ

Canadian Medical Education Journal

EISSN: 1923-1202
Publisher: University of Saskatchewan

ARTICLE INFORMATION
==============================
Subjects: Oe 3–3

Autopsy of a Longitudinal Integrated Clerkship: An Organizational Process Analysis of a Clerkship Program's Demise

Clare Hutchinson University of Toronto

Clare Hutchinson University of Toronto

Abdollah Behzadi University of Toronto

Natalie Clavel University of Toronto

Ra Han University of Toronto

Adam Kaufman University of Toronto

Piero Tartaro University of Toronto

Kulamakan Kulasegaram University of Toronto

Maria Martimianakis University of Toronto

Maria Mylopoulos University of Toronto

Stacey Bernstein University of Toronto

Electronic publication date: 2020 Apr 18

JOURNAL INFORMATION
==============================
NLM Title Abbreviation: Can Med Educ J
Journal ID: Can Med Educ J
NLM Journal ID: 101560935
Journal ID: CMEJ

Canadian Medical Education Journal

EISSN: 1923-1202
Publisher: University of Saskatchewan

ARTICLE INFORMATION
==============================
Subjects: Oe 3–4

Introduction of the National Electives Diversification Policy: A McGill University Case Study

Alexandra Cohen McGill

Sonia Macfarlane McGill

Electronic publication date: 2020 Apr 18

JOURNAL INFORMATION
==============================
NLM Title Abbreviation: Can Med Educ J
Journal ID: Can Med Educ J
NLM Journal ID: 101560935
Journal ID: CMEJ

Canadian Medical Education Journal

EISSN: 1923-1202
Publisher: University of Saskatchewan

ARTICLE INFORMATION
==============================
Subjects: Oe 3–5

The Potential Effect of the Psychiatric Clerkship and Contact-Based Hypothesis on Explicit and Implicit Stigmatizing Attitudes of Canadian Medical Students Towards Mental Illness

Anish Arora McGill

Harman Sandhu McMaster University

Jennifer Brasch McMaster University

Electronic publication date: 2020 Apr 18

JOURNAL INFORMATION
==============================
NLM Title Abbreviation: Can Med Educ J
Journal ID: Can Med Educ J
NLM Journal ID: 101560935
Journal ID: CMEJ

Canadian Medical Education Journal

EISSN: 1923-1202
Publisher: University of Saskatchewan

ARTICLE INFORMATION
==============================
Subjects: Oe 3–6

REACT: A Pre-Clerkship Bootcamp to Improve Student Knowledge and Interest in Critical Care Specialties

Jamie Ghossein University of Ottawa

Vignesh Shankar Sethuraman University of Ottawa

Frank Battaglia University of Ottawa

Neraj Manhas University of Ottawa

Shankar Sethuraman University of Ottawa

Celina DeBiasio University of Ottawa

Rhiannan Pinnell University of Ottawa

Nikhil Rastogi University of Ottawa

Electronic publication date: 2020 Apr 18

JOURNAL INFORMATION
==============================
NLM Title Abbreviation: Can Med Educ J
Journal ID: Can Med Educ J
NLM Journal ID: 101560935
Journal ID: CMEJ

Canadian Medical Education Journal

EISSN: 1923-1202
Publisher: University of Saskatchewan

ARTICLE INFORMATION
==============================
Subjects: Oe 6–5

A modern approach to physician resource planning to improve accessibility and personalized health care for Canadians

Dax Bourcier Université de Sherbrooke

Brandon Collins Memorial University of Newfoundland

Stuti Tanya Memorial University of Newfoundland

Mathieu Doiron Université de Sherbrooke

Natasha Larivée Dalhousie University

Alex Wong Dalhousie University

Marie Vigneau Université de Sherbrooke

Ameer Jarrar Dalhousie University

Victoria Kulesza Dalhousie University

Jelisa Bradley Dalhousie University

Ryan Wade Memorial University of Newfoundland

Patrick Holland Dalhousie University

Electronic publication date: 2020 Apr 18

JOURNAL INFORMATION
==============================
NLM Title Abbreviation: Can Med Educ J
Journal ID: Can Med Educ J
NLM Journal ID: 101560935
Journal ID: CMEJ

Canadian Medical Education Journal

EISSN: 1923-1202
Publisher: University of Saskatchewan

ARTICLE INFORMATION
==============================
Subjects: Oe 6–1

Lost and never found: Exploring the involvement of trainees in the care of people who inject drugs

Lisa Liu Western University

Mark Goldszmidt Western University

Sarah Burm Dalhousie University

Sayra Cristancho Western University

Jacqueline Torti Western University

Javeed Sukhera Western University

Electronic publication date: 2020 Apr 18

JOURNAL INFORMATION
==============================
NLM Title Abbreviation: Can Med Educ J
Journal ID: Can Med Educ J
NLM Journal ID: 101560935
Journal ID: CMEJ

Canadian Medical Education Journal

EISSN: 1923-1202
Publisher: University of Saskatchewan

ARTICLE INFORMATION
==============================
Subjects: Oe 6–2

Creating inclusive spaces in healthcare education: translating knowledge to promote inclusion and equity of people with disabilities in healthcare professions

Yael Mayer University of British Columbia

Tal Jarus University of British Columbia

Laura Bulk University of British Columbia

George Belliveau University of British Columbia

Christopher Cook University of British Columbia

Michael Lee University of British Columbia

Jennica Nichols University of British Columbia

Laen Hershler University of British Columbia

Ally Malinowski University of British Columbia

Yuval Jarus-Hakak University of British Columbia

Emily Gresham University of British Columbia

Electronic publication date: 2020 Apr 18

JOURNAL INFORMATION
==============================
NLM Title Abbreviation: Can Med Educ J
Journal ID: Can Med Educ J
NLM Journal ID: 101560935
Journal ID: CMEJ

Canadian Medical Education Journal

EISSN: 1923-1202
Publisher: University of Saskatchewan

ARTICLE INFORMATION
==============================
Subjects: Oe 6–3

What is a Health Advocate?

Theresa Van Der Goes University of British Columbia

Ian Scott University of British Columbia

Maria Hubinette University of British Columbia

Renate Kahlke The Royal College of Physicians and Surgeons

Electronic publication date: 2020 Apr 18

JOURNAL INFORMATION
==============================
NLM Title Abbreviation: Can Med Educ J
Journal ID: Can Med Educ J
NLM Journal ID: 101560935
Journal ID: CMEJ

Canadian Medical Education Journal

EISSN: 1923-1202
Publisher: University of Saskatchewan

ARTICLE INFORMATION
==============================
Subjects: Oe 6–4

Connecting lived experience with medical students: A pen-pal curriculum innovation to promote compassion regarding mental health and homelessness

Jackie Tsang University of Toronto

Ivona Berger University of Toronto

Abirami Kirubarajan University of Toronto

Seiwon Park University of Toronto

Roxanne Wright University of Toronto

Carmen Charles University of Toronto

Judith Marshall University of Toronto

Fok-Han Leung University of Toronto

Electronic publication date: 2020 Apr 18

JOURNAL INFORMATION
==============================
NLM Title Abbreviation: Can Med Educ J
Journal ID: Can Med Educ J
NLM Journal ID: 101560935
Journal ID: CMEJ

Canadian Medical Education Journal

EISSN: 1923-1202
Publisher: University of Saskatchewan

ARTICLE INFORMATION
==============================
Subjects: Oe 6–6

Transforming our relationship with the social determinants of health: a scoping review of social justice interventions in Canadian medical schools

Nisha Kansal McMaster University

Anvita Kulkarni Queen’s University

Janice Lee University of Toronto

Brittany Graham McMaster University

Michael Kruse McMaster University

Megan Chu McMaster University

Sureka Pavalagantharajah McMaster University

Jason Profetto McMaster University

Albina Veltman McMaster University

Electronic publication date: 2020 Apr 18

JOURNAL INFORMATION
==============================
NLM Title Abbreviation: Can Med Educ J
Journal ID: Can Med Educ J
NLM Journal ID: 101560935
Journal ID: CMEJ

Canadian Medical Education Journal

EISSN: 1923-1202
Publisher: University of Saskatchewan

ARTICLE INFORMATION
==============================
Subjects: Oe 4–1

Canadian Program Directors' Perceptions of Unmatched Canadian Medical Graduates

K. Taneille Johnson University of British Columbia

Alex Dotto University of British Columbia

Aalok Kumar University of British Columbia

Carol-Ann Courneya University of British Columbia

Electronic publication date: 2020 Apr 18

JOURNAL INFORMATION
==============================
NLM Title Abbreviation: Can Med Educ J
Journal ID: Can Med Educ J
NLM Journal ID: 101560935
Journal ID: CMEJ

Canadian Medical Education Journal

EISSN: 1923-1202
Publisher: University of Saskatchewan

ARTICLE INFORMATION
==============================
Subjects: Oe 4–2

Analysis of Factors Affecting Canadian Medical Students' Success in the Residency Match

Kelly Howse Queen’s University

Nicholas Cofie Queen’s University

Nancy Dalgarno Queen’s University

Joshua Lakoff Queen’s University

Electronic publication date: 2020 Apr 18

JOURNAL INFORMATION
==============================
NLM Title Abbreviation: Can Med Educ J
Journal ID: Can Med Educ J
NLM Journal ID: 101560935
Journal ID: CMEJ

Canadian Medical Education Journal

EISSN: 1923-1202
Publisher: University of Saskatchewan

ARTICLE INFORMATION
==============================
Subjects: Oe 4–3

Benefits and challenges of residency remediation programs: perspective of former remediated residents

Sara Turcotte McGill

Rosario (Charo) Rodriguez McGill

Fok-Han Leung University of Toronto

Milena Forte University of Toronto

Kathleen Rice McGill

Perle Feldman McGill

Electronic publication date: 2020 Apr 18

JOURNAL INFORMATION
==============================
NLM Title Abbreviation: Can Med Educ J
Journal ID: Can Med Educ J
NLM Journal ID: 101560935
Journal ID: CMEJ

Canadian Medical Education Journal

EISSN: 1923-1202
Publisher: University of Saskatchewan

ARTICLE INFORMATION
==============================
Subjects: Oe 4–4

The Socioeconomic Cost of Residency Application in Canada

Assil Abda Université de Montréal

David-Dan Nguyen McGill

Sarah Mecheddal Université de Montréal

Marco Mascarella McGill

Lily Nguyen McGill

Electronic publication date: 2020 Apr 18

JOURNAL INFORMATION
==============================
NLM Title Abbreviation: Can Med Educ J
Journal ID: Can Med Educ J
NLM Journal ID: 101560935
Journal ID: CMEJ

Canadian Medical Education Journal

EISSN: 1923-1202
Publisher: University of Saskatchewan

ARTICLE INFORMATION
==============================
Subjects: Oe 4–5

Exploring the construct of anticipatory stress and finding a job after residency training

Aliya Kassam University of Calgary

Megan Thomas University of Calgary

Kent Hecker University of Calgary

Electronic publication date: 2020 Apr 18

JOURNAL INFORMATION
==============================
NLM Title Abbreviation: Can Med Educ J
Journal ID: Can Med Educ J
NLM Journal ID: 101560935
Journal ID: CMEJ

Canadian Medical Education Journal

EISSN: 1923-1202
Publisher: University of Saskatchewan

ARTICLE INFORMATION
==============================
Subjects: Oe 4–6

Residents' Motivation in Clinical Settings and Exam Performance: Unexpected Findings

Janelle Sloychuk University of Alberta

Oksana Babenko University of Alberta

Olga Szafran University of Alberta

Kimberley Duerksen University of Alberta

Electronic publication date: 2020 Apr 18

JOURNAL INFORMATION
==============================
NLM Title Abbreviation: Can Med Educ J
Journal ID: Can Med Educ J
NLM Journal ID: 101560935
Journal ID: CMEJ

Canadian Medical Education Journal

EISSN: 1923-1202
Publisher: University of Saskatchewan

ARTICLE INFORMATION
==============================
Subjects: Oe 5–1

A Rural University-High School Healthcare Career Community Engagement Initiative: The Healthcare Travelling Roadshow.

Sean Maurice University of Northern British Columbia

Kristjan Mytting University of Northern British Columbia

Quinn Gentles University of British Columbia

Robin Roots University of British Columbia

Alina Constantin University of Northern British Columbia

Sonya Kruger University of Northern British Columbia

Warren Brock University of British Columbia

Olusegun Oyedele University of British Columbia

John Soles District of Clearwater

Shelley Sim District of Clearwater

David Snadden University of British Columbia

Electronic publication date: 2020 Apr 18

JOURNAL INFORMATION
==============================
NLM Title Abbreviation: Can Med Educ J
Journal ID: Can Med Educ J
NLM Journal ID: 101560935
Journal ID: CMEJ

Canadian Medical Education Journal

EISSN: 1923-1202
Publisher: University of Saskatchewan

ARTICLE INFORMATION
==============================
Subjects: Oe 5–2

Understanding gender-based needs in rural physician mentoring programs

Stephanie Gariscsak University of British Columbia

Jenna Lightbody University of British Columbia

Bob Bluman University of British Columbia

Brenna Lynn University of British Columbia

Electronic publication date: 2020 Apr 18

JOURNAL INFORMATION
==============================
NLM Title Abbreviation: Can Med Educ J
Journal ID: Can Med Educ J
NLM Journal ID: 101560935
Journal ID: CMEJ

Canadian Medical Education Journal

EISSN: 1923-1202
Publisher: University of Saskatchewan

ARTICLE INFORMATION
==============================
Subjects: Oe 5–3

Driving Socially Accountable Medical Education through a Research Program for Rural Faculty

Wendy Graham Memorial University of Newfoundland

Thomas Heeley Memorial University of Newfoundland

Shabnam Asghari Memorial University of Newfoundland

Cheri Bethune Memorial – University of Newfoundland

Electronic publication date: 2020 Apr 18

JOURNAL INFORMATION
==============================
NLM Title Abbreviation: Can Med Educ J
Journal ID: Can Med Educ J
NLM Journal ID: 101560935
Journal ID: CMEJ

Canadian Medical Education Journal

EISSN: 1923-1202
Publisher: University of Saskatchewan

ARTICLE INFORMATION
==============================
Subjects: Oe 5–4

Improving Resident Education and Patient Care through National Physician Licensure

Brandon Tang University of British Columbia

Bernard Ho University of Toronto

Electronic publication date: 2020 Apr 18

JOURNAL INFORMATION
==============================
NLM Title Abbreviation: Can Med Educ J
Journal ID: Can Med Educ J
NLM Journal ID: 101560935
Journal ID: CMEJ

Canadian Medical Education Journal

EISSN: 1923-1202
Publisher: University of Saskatchewan

ARTICLE INFORMATION
==============================
Subjects: Oe 5–5

“You can't address the other stories until you address the big ones”: Insights from rural practitioner palliative care learning

Frances Kilbertus Northern Ontario School of Medicine

King Keely Northern Ontario School of Medicine

Susan Robinson Northern Ontario School of Medicine

Sayra Cristancho Western University

Sarah Burm Dalhousie University

Electronic publication date: 2020 Apr 18

JOURNAL INFORMATION
==============================
NLM Title Abbreviation: Can Med Educ J
Journal ID: Can Med Educ J
NLM Journal ID: 101560935
Journal ID: CMEJ

Canadian Medical Education Journal

EISSN: 1923-1202
Publisher: University of Saskatchewan

ARTICLE INFORMATION
==============================
Subjects: Oe 5–6

Mixed Messages: A Visual and Textual Analysis of a Rural Medicine Website

Rebecca Malhi University of Calgary

Douglas Myhre University of Calgary

Electronic publication date: 2020 Apr 18

JOURNAL INFORMATION
==============================
NLM Title Abbreviation: Can Med Educ J
Journal ID: Can Med Educ J
NLM Journal ID: 101560935
Journal ID: CMEJ

Canadian Medical Education Journal

EISSN: 1923-1202
Publisher: University of Saskatchewan

ARTICLE INFORMATION
==============================
Subjects: Oe 7–1

Evaluating an Integrated Ethics Curriculum: Evidence of Process and Outcome Changes

Kulamakan Kulasegaram University of Toronto

Carrie Bernard University of Toronto

Betty Onyura University of Toronto

Eva Knifed University of Toronto

Erika Abner University of Toronto

Nadia Incardona University of Toronto

Irene Ying University of Toronto

Frank Wagner University of Toronto

Risa Freeman University of Toronto

Electronic publication date: 2020 Apr 18

JOURNAL INFORMATION
==============================
NLM Title Abbreviation: Can Med Educ J
Journal ID: Can Med Educ J
NLM Journal ID: 101560935
Journal ID: CMEJ

Canadian Medical Education Journal

EISSN: 1923-1202
Publisher: University of Saskatchewan

ARTICLE INFORMATION
==============================
Subjects: Oe 7–2

Residents as research subjects: balancing resident education with contribution to advancing educational innovations.

Louis-Philippe Thibault-Lemyre Université de Montréal

Ahmed Moussa Université de Montréal

Thuy Mai Luu Université de Montréal

Celine Huot Université de Montréal

Genevieve Cardinal Université de Montréal

Benoit Carriere Université de Montréal

Electronic publication date: 2020 Apr 18

JOURNAL INFORMATION
==============================
NLM Title Abbreviation: Can Med Educ J
Journal ID: Can Med Educ J
NLM Journal ID: 101560935
Journal ID: CMEJ

Canadian Medical Education Journal

EISSN: 1923-1202
Publisher: University of Saskatchewan

ARTICLE INFORMATION
==============================
Subjects: Oe 7–3

Using the Francophone Literature of Western Canada to Weave Humanism into the Fabric of Medical Education

Jan Marta University of Toronto

Electronic publication date: 2020 Apr 18

JOURNAL INFORMATION
==============================
NLM Title Abbreviation: Can Med Educ J
Journal ID: Can Med Educ J
NLM Journal ID: 101560935
Journal ID: CMEJ

Canadian Medical Education Journal

EISSN: 1923-1202
Publisher: University of Saskatchewan

ARTICLE INFORMATION
==============================
Subjects: Oe 7–4

A scoping review on the uses of the arts and humanities in medical education

Tracy Moniz Mount Saint Vincent University

Maryam Golafshani University of Toronto

Lorelei Lingard Western University

Paul Haidet Penn State College of Medicine

Nancy E. Adams Penn State College of Medicine

Javeed Sukhera Western University

Carolyn Gaspar Dalhousie University

Rebecca Volpe Penn State College of Medicine

Claire De Boer Penn State College of Medicine

Tavis Apramian Western University

Shannon Arntfield Western University

Electronic publication date: 2020 Apr 18

JOURNAL INFORMATION
==============================
NLM Title Abbreviation: Can Med Educ J
Journal ID: Can Med Educ J
NLM Journal ID: 101560935
Journal ID: CMEJ

Canadian Medical Education Journal

EISSN: 1923-1202
Publisher: University of Saskatchewan

ARTICLE INFORMATION
==============================
Subjects: Oe 7–5

Educating for patient centered end of life care: Understanding the temporal, occupational, and relational dimensions that shape dying patients' experiences

Laura Yvonne Bulk University of British Columbia

Laura Nimmon University of British Columbia

Gil Kimel University of British Columbia

Nigel King University of Huddersfield

Electronic publication date: 2020 Apr 18

JOURNAL INFORMATION
==============================
NLM Title Abbreviation: Can Med Educ J
Journal ID: Can Med Educ J
NLM Journal ID: 101560935
Journal ID: CMEJ

Canadian Medical Education Journal

EISSN: 1923-1202
Publisher: University of Saskatchewan

ARTICLE INFORMATION
==============================
Subjects: Oe 7–6

Informing a medical assistance in dying curriculum in specialty residency training programs

Susan MacDonald Queen’s University

Nancy Dalgarno Queen’s University

Mary Martin Queen’s University

Sarah LeBlanc Queen’s University

Ross Walker Queen’s University

David Taylor Queen’s University

Karen Smith Queen’s University

Richard Van Wylick Queen’s University

Rylan Egan Queen’s University

Karen Schultz Queen’s University

Electronic publication date: 2020 Apr 18

JOURNAL INFORMATION
==============================
NLM Title Abbreviation: Can Med Educ J
Journal ID: Can Med Educ J
NLM Journal ID: 101560935
Journal ID: CMEJ

Canadian Medical Education Journal

EISSN: 1923-1202
Publisher: University of Saskatchewan

ARTICLE INFORMATION
==============================
Subjects: Of 1–1

Contagion: An innovative video-game based approach to antimicrobial stewardship and education

Samveg Shah McMaster University

Maroof Khalid McMaster University

Sudarshan Bala McMaster University

Maynard Luterman McMaster University

Yasmeen Vincent McMaster University

Electronic publication date: 2020 Apr 18

JOURNAL INFORMATION
==============================
NLM Title Abbreviation: Can Med Educ J
Journal ID: Can Med Educ J
NLM Journal ID: 101560935
Journal ID: CMEJ

Canadian Medical Education Journal

EISSN: 1923-1202
Publisher: University of Saskatchewan

ARTICLE INFORMATION
==============================
Subjects: Of 1–2

CARTOONING AND DRUG USE: Creating Art for Stigmatized Topics

Armin Mortazavi University of British Columbia

Kate Campbell University of British Columbia

Andrea Keesey University of British Columbia

Brenna Lynn University of British Columbia

Electronic publication date: 2020 Apr 18

JOURNAL INFORMATION
==============================
NLM Title Abbreviation: Can Med Educ J
Journal ID: Can Med Educ J
NLM Journal ID: 101560935
Journal ID: CMEJ

Canadian Medical Education Journal

EISSN: 1923-1202
Publisher: University of Saskatchewan

ARTICLE INFORMATION
==============================
Subjects: Of 1–3

Theatre as an innovative educational tool in health professions education

Tal Jarus University of British Columbia

Yael Mayer University of British Columbia

Laura Bulk University of British Columbia

Laen Hershier University of British Columbia

Christopher Cook University of British Columbia

Jennica Nichols University of British Columbia

George Belliveau University of British Columbia

Michael Lee University of British Columbia

Electronic publication date: 2020 Apr 18

JOURNAL INFORMATION
==============================
NLM Title Abbreviation: Can Med Educ J
Journal ID: Can Med Educ J
NLM Journal ID: 101560935
Journal ID: CMEJ

Canadian Medical Education Journal

EISSN: 1923-1202
Publisher: University of Saskatchewan

ARTICLE INFORMATION
==============================
Subjects: Of 1–4

Bringing the Patient Voice to Professionalism in Medical Education

Simon Haney University of Toronto

Shiphra Ginsburg University of Toronto

Ayelet Kuper University of Toronto

Ryan Brydges University of Toronto

Paula Rowland University of Toronto

Electronic publication date: 2020 Apr 18

JOURNAL INFORMATION
==============================
NLM Title Abbreviation: Can Med Educ J
Journal ID: Can Med Educ J
NLM Journal ID: 101560935
Journal ID: CMEJ

Canadian Medical Education Journal

EISSN: 1923-1202
Publisher: University of Saskatchewan

ARTICLE INFORMATION
==============================
Subjects: Of 1–5

Three applications of learning sciences in medical education

Tracey Hillier University of Alberta

Doris Lunardon University of Alberta

Vijay Daniels University of Alberta

Anna Oswald University of Alberta

Cody Surgin University of Alberta

Hollis Lai University of Alberta

Electronic publication date: 2020 Apr 18

JOURNAL INFORMATION
==============================
NLM Title Abbreviation: Can Med Educ J
Journal ID: Can Med Educ J
NLM Journal ID: 101560935
Journal ID: CMEJ

Canadian Medical Education Journal

EISSN: 1923-1202
Publisher: University of Saskatchewan

ARTICLE INFORMATION
==============================
Subjects: Of 1–6

Discovery Healthcare: Encouraging Highschool Students in South-Western Ontario to Pursue Careers in Healthcare

Arita Alija Western University

Julia Petta Western University

Richard Yu Western University

Vivian Tia Western University

Electronic publication date: 2020 Apr 18

JOURNAL INFORMATION
==============================
NLM Title Abbreviation: Can Med Educ J
Journal ID: Can Med Educ J
NLM Journal ID: 101560935
Journal ID: CMEJ

Canadian Medical Education Journal

EISSN: 1923-1202
Publisher: University of Saskatchewan

ARTICLE INFORMATION
==============================
Subjects: Of 2–1

Evaluation of the Indigenous Health Curriculum in Canadian Undergraduate Medical Education

Muskaan Sachdeva University of Toronto

Lisa Richardson University of Toronto

Cynthia Whitehead University of Toronto

Robert Paul University of Toronto

Electronic publication date: 2020 Apr 18

JOURNAL INFORMATION
==============================
NLM Title Abbreviation: Can Med Educ J
Journal ID: Can Med Educ J
NLM Journal ID: 101560935
Journal ID: CMEJ

Canadian Medical Education Journal

EISSN: 1923-1202
Publisher: University of Saskatchewan

ARTICLE INFORMATION
==============================
Subjects: Of 2–2

Adaptation and Adoption: How Trainees Make Sense of Clinical Practice Variation

Katherine Ng University of British Columbia

Kevin Eva University of British Columbia

Luke Chen University of British Columbia

Electronic publication date: 2020 Apr 18

JOURNAL INFORMATION
==============================
NLM Title Abbreviation: Can Med Educ J
Journal ID: Can Med Educ J
NLM Journal ID: 101560935
Journal ID: CMEJ

Canadian Medical Education Journal

EISSN: 1923-1202
Publisher: University of Saskatchewan

ARTICLE INFORMATION
==============================
Subjects: Of 2–3

Une nouvelle fiche d'évaluation au service de l'évaluation en APC : l'expérience de l'Université de Montréal

Diane Robert Université de Montréal

Marie-Pierre Codsi Université de Montréal

Isabelle Gosselin Université de Montréal

Samuel Gatien Université de Montréal

Electronic publication date: 2020 Apr 18

JOURNAL INFORMATION
==============================
NLM Title Abbreviation: Can Med Educ J
Journal ID: Can Med Educ J
NLM Journal ID: 101560935
Journal ID: CMEJ

Canadian Medical Education Journal

EISSN: 1923-1202
Publisher: University of Saskatchewan

ARTICLE INFORMATION
==============================
Subjects: Of 2–4

An Innovative e-Prescribing Learning Platform for Medical Students and Residents

Marie Rocchi University of Toronto

Janet Cooper Association of Faculties of Pharmacy of Canada

Electronic publication date: 2020 Apr 18

JOURNAL INFORMATION
==============================
NLM Title Abbreviation: Can Med Educ J
Journal ID: Can Med Educ J
NLM Journal ID: 101560935
Journal ID: CMEJ

Canadian Medical Education Journal

EISSN: 1923-1202
Publisher: University of Saskatchewan

ARTICLE INFORMATION
==============================
Subjects: Of 2–5

Reading of the Week - A Novel (and National) Education Project for Psychiatry Residents

David Gratzer University of Toronto

Faisal Islam McMaster University

Electronic publication date: 2020 Apr 18

JOURNAL INFORMATION
==============================
NLM Title Abbreviation: Can Med Educ J
Journal ID: Can Med Educ J
NLM Journal ID: 101560935
Journal ID: CMEJ

Canadian Medical Education Journal

EISSN: 1923-1202
Publisher: University of Saskatchewan

ARTICLE INFORMATION
==============================
Subjects: Of 2–6

Digital Tattoo - A Workshop to Support Student Understanding of the Impact of Social Media Platforms

Patricia Gerber University of British Columbia

Alexandra Kuskowski , University of British Columbia

Kathleen Scheaffer University of Toronto

Lucas Wright University of British Columbia

Salma Abumeeiz University of British Columbia

Laura Atiyeh University of British Columbia

Emily Fornwald University of British Columbia

Ursula Ellis University of British Columbia

Eseohe Ojo University of British Columbia

Electronic publication date: 2020 Apr 18

JOURNAL INFORMATION
==============================
NLM Title Abbreviation: Can Med Educ J
Journal ID: Can Med Educ J
NLM Journal ID: 101560935
Journal ID: CMEJ

Canadian Medical Education Journal

EISSN: 1923-1202
Publisher: University of Saskatchewan

ARTICLE INFORMATION
==============================
Subjects: Of 3–1

Tweet this! Using Social Media as an Innovative Tool for Increasing Survey Responses

Maryam Wagner McGill

Jackie Roberge-Dao McGill

Aliki Thomas McGill

Monica Slanik McGill

Bernadette Nedelec McGill

Cynthia Perlman McGill

Sara Saunders McGill

Electronic publication date: 2020 Apr 18

JOURNAL INFORMATION
==============================
NLM Title Abbreviation: Can Med Educ J
Journal ID: Can Med Educ J
NLM Journal ID: 101560935
Journal ID: CMEJ

Canadian Medical Education Journal

EISSN: 1923-1202
Publisher: University of Saskatchewan

ARTICLE INFORMATION
==============================
Subjects: Of 3–2

An innovative approach to Program Evaluation in PGME: Design, Development, and Implementation

Theresa Beesley McGill

Carlos Gomez-Garibello McGill

Maryam Wagner McGill

Evelyn Constantin McGill

Regina Husa McGill

Armand Aalamian McGill

Electronic publication date: 2020 Apr 18

JOURNAL INFORMATION
==============================
NLM Title Abbreviation: Can Med Educ J
Journal ID: Can Med Educ J
NLM Journal ID: 101560935
Journal ID: CMEJ

Canadian Medical Education Journal

EISSN: 1923-1202
Publisher: University of Saskatchewan

ARTICLE INFORMATION
==============================
Subjects: Of 3–3

Developing an evaluation plan for CBME by capturing the interests of program directors across specialties

Deena Hamza University of Alberta

Anna Oswald University of Alberta

Electronic publication date: 2020 Apr 18

JOURNAL INFORMATION
==============================
NLM Title Abbreviation: Can Med Educ J
Journal ID: Can Med Educ J
NLM Journal ID: 101560935
Journal ID: CMEJ

Canadian Medical Education Journal

EISSN: 1923-1202
Publisher: University of Saskatchewan

ARTICLE INFORMATION
==============================
Subjects: Of 3–4

The CanadiEM Junior Editor Program: A quantitative study and program evaluation

Sonja Wakeling McMaster University

Brent Thoma University of Saskatchewan

Teresa Chan McMaster University

Electronic publication date: 2020 Apr 18

JOURNAL INFORMATION
==============================
NLM Title Abbreviation: Can Med Educ J
Journal ID: Can Med Educ J
NLM Journal ID: 101560935
Journal ID: CMEJ

Canadian Medical Education Journal

EISSN: 1923-1202
Publisher: University of Saskatchewan

ARTICLE INFORMATION
==============================
Subjects: Of 3–5

How are postgraduate medical educators using reflective writing to remediate professionalism? A constructivist grounded theory study

Tracy Moniz Mount Saint Vincent University

Carolyn Gaspar Dalhousie University

Andrew Warren Dalhousie University

Chris Watling Western University

Electronic publication date: 2020 Apr 18

JOURNAL INFORMATION
==============================
NLM Title Abbreviation: Can Med Educ J
Journal ID: Can Med Educ J
NLM Journal ID: 101560935
Journal ID: CMEJ

Canadian Medical Education Journal

EISSN: 1923-1202
Publisher: University of Saskatchewan

ARTICLE INFORMATION
==============================
Subjects: Of 3–6

The Nature of Wisdom in Clinical Medicine

Donald Boudreau McGill

Eric Cassell Weill Medical College of Cornell University

Electronic publication date: 2020 Apr 18

JOURNAL INFORMATION
==============================
NLM Title Abbreviation: Can Med Educ J
Journal ID: Can Med Educ J
NLM Journal ID: 101560935
Journal ID: CMEJ

Canadian Medical Education Journal

EISSN: 1923-1202
Publisher: University of Saskatchewan

ARTICLE INFORMATION
==============================
Subjects: Of 6–1

Medical trainees' push for cultural competence through internships with neglected communities

Rosa Lakabi McGill

Sarah Chibane McGill

Electronic publication date: 2020 Apr 18

JOURNAL INFORMATION
==============================
NLM Title Abbreviation: Can Med Educ J
Journal ID: Can Med Educ J
NLM Journal ID: 101560935
Journal ID: CMEJ

Canadian Medical Education Journal

EISSN: 1923-1202
Publisher: University of Saskatchewan

ARTICLE INFORMATION
==============================
Subjects: Of 6–2

Trends in the proportion of female provincial and national residency organization award recipients across Canada from 2000 to 2018

Sarah Silverberg University of British Columbia

Shannon Ruzycki University of Calgary

Irene Ma University of Calgary

Electronic publication date: 2020 Apr 18

JOURNAL INFORMATION
==============================
NLM Title Abbreviation: Can Med Educ J
Journal ID: Can Med Educ J
NLM Journal ID: 101560935
Journal ID: CMEJ

Canadian Medical Education Journal

EISSN: 1923-1202
Publisher: University of Saskatchewan

ARTICLE INFORMATION
==============================
Subjects: Of 6–3

Preparing service providers to 'champion' translation of cultural safety and trauma-informed care into service delivery and practice

Kimberly Miller Sunny Hill Health Centre for Children

Stephanie Glegg Sunny Hill Health Centre for Children

Jason Gordon BC Association for Child Development and Intervention

Electronic publication date: 2020 Apr 18

JOURNAL INFORMATION
==============================
NLM Title Abbreviation: Can Med Educ J
Journal ID: Can Med Educ J
NLM Journal ID: 101560935
Journal ID: CMEJ

Canadian Medical Education Journal

EISSN: 1923-1202
Publisher: University of Saskatchewan

ARTICLE INFORMATION
==============================
Subjects: Of 6–4

Créer un lieu d'échange culturel, interdisciplinaire et intellectuel entre les étudiants et étudiantes et les membres des Premières Nations : les mini-écoles en sciences de la santé de l'Université Laval

Henri Cyr Université Laval

Sonia Jalbert Université Laval

Emmanuelle Careau Université Laval

Dominique Vandal Université Laval

Electronic publication date: 2020 Apr 18

JOURNAL INFORMATION
==============================
NLM Title Abbreviation: Can Med Educ J
Journal ID: Can Med Educ J
NLM Journal ID: 101560935
Journal ID: CMEJ

Canadian Medical Education Journal

EISSN: 1923-1202
Publisher: University of Saskatchewan

ARTICLE INFORMATION
==============================
Subjects: Of 6–5

Developing a New Measure of Cultural Sensitivity for Health Professions Learners: Better Metrics for Observerships

Rylan Egan Queen’s University

Nazik Hammad Queen’s University

Eleftherios Soleas Queen’s University

Jennifer Carpenter Queen’s University

Mikaila De sousa Queen’s University

Nicholas Cofie Queen’s University

Electronic publication date: 2020 Apr 18

JOURNAL INFORMATION
==============================
NLM Title Abbreviation: Can Med Educ J
Journal ID: Can Med Educ J
NLM Journal ID: 101560935
Journal ID: CMEJ

Canadian Medical Education Journal

EISSN: 1923-1202
Publisher: University of Saskatchewan

ARTICLE INFORMATION
==============================
Subjects: Of 6–6

Later is too late: Exploring student experiences of diversity and inclusion in medical school orientation

Wid Yaseen University of Toronto

Asia van Buuren University of Toronto

Paula Veinot Independent Research Consultant, Halifax, Nova Scotia, Canada

Maria Mylopoulos University of Toronto

Marcus Law University of Toronto

Electronic publication date: 2020 Apr 18

JOURNAL INFORMATION
==============================
NLM Title Abbreviation: Can Med Educ J
Journal ID: Can Med Educ J
NLM Journal ID: 101560935
Journal ID: CMEJ

Canadian Medical Education Journal

EISSN: 1923-1202
Publisher: University of Saskatchewan

ARTICLE INFORMATION
==============================
Subjects: Of 4–1

Développement du rôle d'éducateur des étudiants en médecine dans un programme fondé sur une approche par compétence.

Paul Chiasson Université de Sherbrooke

Louis Gagnon Université de Sherbrooke

Sylvie Mathieu Université de Sherbrooke

Sylvie Houde Université de Sherbrooke

Cécile Trochet Université de Sherbrooke

Nathalie Bettez Université de Sherbrooke

Marie-Josée Leblanc Université de Sherbrooke

Frédéric Bernier Université de Sherbrooke

Ghislaine Houde Université de Sherbrooke

Ann Graillon Université de Sherbrooke

Evelyne Cambron-Goulet Université de Sherbrooke

Electronic publication date: 2020 Apr 18

JOURNAL INFORMATION
==============================
NLM Title Abbreviation: Can Med Educ J
Journal ID: Can Med Educ J
NLM Journal ID: 101560935
Journal ID: CMEJ

Canadian Medical Education Journal

EISSN: 1923-1202
Publisher: University of Saskatchewan

ARTICLE INFORMATION
==============================
Subjects: Of 4–2

Advancing Scholarly work across Ontario's Distributed Medical Education programs

Larry Chambers McMaster University

Alison Freeland University of Toronto

Charles Su University of Ottawa

Dorothy Bakker McMaster University

Ruzica Jokic Queen’s University

George Kim Western University

Electronic publication date: 2020 Apr 18

JOURNAL INFORMATION
==============================
NLM Title Abbreviation: Can Med Educ J
Journal ID: Can Med Educ J
NLM Journal ID: 101560935
Journal ID: CMEJ

Canadian Medical Education Journal

EISSN: 1923-1202
Publisher: University of Saskatchewan

ARTICLE INFORMATION
==============================
Subjects: Of 4–3

Development of the role of researcher of medical students in a program based on a competency-based approach.

Mathieu Bélanger Université de Sherbrooke

Luigi Bouchard Université de Sherbrooke

Fanny Lapointe Université de Sherbrooke

Frédéric Bernier Université de Sherbrooke

Ghislaine Houde Université de Sherbrooke

Paul Farand Université de Sherbrooke

Electronic publication date: 2020 Apr 18

JOURNAL INFORMATION
==============================
NLM Title Abbreviation: Can Med Educ J
Journal ID: Can Med Educ J
NLM Journal ID: 101560935
Journal ID: CMEJ

Canadian Medical Education Journal

EISSN: 1923-1202
Publisher: University of Saskatchewan

ARTICLE INFORMATION
==============================
Subjects: Of 4–4

Application of the Holmes' Reflection Model to Promote Professionalism in Medical Students upon Starting Clinical Rotations: A Randomized Controlled Trial

Sara Mortaz Hejri McGill

Leila Naimi Tehran University of Medical Sciences

Electronic publication date: 2020 Apr 18

JOURNAL INFORMATION
==============================
NLM Title Abbreviation: Can Med Educ J
Journal ID: Can Med Educ J
NLM Journal ID: 101560935
Journal ID: CMEJ

Canadian Medical Education Journal

EISSN: 1923-1202
Publisher: University of Saskatchewan

ARTICLE INFORMATION
==============================
Subjects: Of 4–5

Navigating Ottawa Resources to Improve Health: An Evaluation of a Multidisciplinary Ottawa Student-Run Clinic

Maria Merlano University of Ottawa

Julie Bain University of Ottawa

Michael Hirsh University of Ottawa

Katina Zheng University of Ottawa

Electronic publication date: 2020 Apr 18

JOURNAL INFORMATION
==============================
NLM Title Abbreviation: Can Med Educ J
Journal ID: Can Med Educ J
NLM Journal ID: 101560935
Journal ID: CMEJ

Canadian Medical Education Journal

EISSN: 1923-1202
Publisher: University of Saskatchewan

ARTICLE INFORMATION
==============================
Subjects: Of 4–6

Physician Leadership Explored from the Perspective of Medical Students

Albert Vo Western University

Jacqueline Torti Western University

Nabil Sultan Western University

Electronic publication date: 2020 Apr 18

JOURNAL INFORMATION
==============================
NLM Title Abbreviation: Can Med Educ J
Journal ID: Can Med Educ J
NLM Journal ID: 101560935
Journal ID: CMEJ

Canadian Medical Education Journal

EISSN: 1923-1202
Publisher: University of Saskatchewan

ARTICLE INFORMATION
==============================
Subjects: Of 5–4

Medical Student Mistreatment and Reporting: A Journey

Amanda Bell McMaster University

Catherine Connelly McMaster University

Allyn Walsh McMaster University

Meredith Vanstone McMaster University

Electronic publication date: 2020 Apr 18

JOURNAL INFORMATION
==============================
NLM Title Abbreviation: Can Med Educ J
Journal ID: Can Med Educ J
NLM Journal ID: 101560935
Journal ID: CMEJ

Canadian Medical Education Journal

EISSN: 1923-1202
Publisher: University of Saskatchewan

ARTICLE INFORMATION
==============================
Subjects: Of 5–2

Risks, Responsibility, and Resiliency: Qualitative Insights on Accessing Mental Health Care and Maintaining Wellness in Ontario Medical Students

Wendy (Wen Qing) Ye McMaster University

Bradley Rietze Northern Ontario School of Medicine

Sydney McQueen University of Toronto

Lena Quilty Centre for Addiction and Mental Health (CAMH)

Christine Wickens University of Toronto

Electronic publication date: 2020 Apr 18

JOURNAL INFORMATION
==============================
NLM Title Abbreviation: Can Med Educ J
Journal ID: Can Med Educ J
NLM Journal ID: 101560935
Journal ID: CMEJ

Canadian Medical Education Journal

EISSN: 1923-1202
Publisher: University of Saskatchewan

ARTICLE INFORMATION
==============================
Subjects: Of 5–3

Improving Learner Wellbeing: A Two year review of the CFMS National Wellness Program (NWP)

Misha Virdee McMaster University

Dax Bourcier Université de Sherbrooke

Victor Do University of Alberta

Electronic publication date: 2020 Apr 18

JOURNAL INFORMATION
==============================
NLM Title Abbreviation: Can Med Educ J
Journal ID: Can Med Educ J
NLM Journal ID: 101560935
Journal ID: CMEJ

Canadian Medical Education Journal

EISSN: 1923-1202
Publisher: University of Saskatchewan

ARTICLE INFORMATION
==============================
Subjects: Of 5–1

Medical Students' Reasons to Not Report Mistreatment

Namta Gupta McGill

Léanne Roncière McGill

Electronic publication date: 2020 Apr 18

JOURNAL INFORMATION
==============================
NLM Title Abbreviation: Can Med Educ J
Journal ID: Can Med Educ J
NLM Journal ID: 101560935
Journal ID: CMEJ

Canadian Medical Education Journal

EISSN: 1923-1202
Publisher: University of Saskatchewan

ARTICLE INFORMATION
==============================
Subjects: Of 5–5

Moral Distress Among Critical Care Physicians: Implications for Medical Education

Dominique Piquette University of Toronto

Aimee Sarti University of Ottawa

Franco Carnevale McGill

Karen Burns University of Toronto

Peter Dodek University of British Columbia

Electronic publication date: 2020 Apr 18

JOURNAL INFORMATION
==============================
NLM Title Abbreviation: Can Med Educ J
Journal ID: Can Med Educ J
NLM Journal ID: 101560935
Journal ID: CMEJ

Canadian Medical Education Journal

EISSN: 1923-1202
Publisher: University of Saskatchewan

ARTICLE INFORMATION
==============================
Subjects: Of 5–6

The fatigue paradox: a grounded theory study of physicians', nurses' and residents' perceptions of physician fatigue

Taryn Taylor Western University

Lorelei Lingard Western University

Sandra Deluca Western University

Julie Ann Vankoughnett Western University

Richard Cherry Western University

Emily Field Western University

Electronic publication date: 2020 Apr 18

JOURNAL INFORMATION
==============================
NLM Title Abbreviation: Can Med Educ J
Journal ID: Can Med Educ J
NLM Journal ID: 101560935
Journal ID: CMEJ

Canadian Medical Education Journal

EISSN: 1923-1202
Publisher: University of Saskatchewan

ARTICLE INFORMATION
==============================
Subjects: Of 7–1

Recognizing our Hidden Faculty: 3-year outcomes of the Health Professional Educators (HPE) program in the Department of Family and Community Medicine at the University of Toronto

Deborah Kopansky-Giles University of Toronto

Judith Peranson University of Toronto

Electronic publication date: 2020 Apr 18

JOURNAL INFORMATION
==============================
NLM Title Abbreviation: Can Med Educ J
Journal ID: Can Med Educ J
NLM Journal ID: 101560935
Journal ID: CMEJ

Canadian Medical Education Journal

EISSN: 1923-1202
Publisher: University of Saskatchewan

ARTICLE INFORMATION
==============================
Subjects: Of 7–2

Rethinking “The [Past] Medical History”: An Exploration of Patient Networks of Care Providers

Laurent Perrault-Sequeira Western University

Jacqueline Torti Western University

Andrew Appleton Western University

Maria Mathews Western University

Mark Goldszmidt Western University

Electronic publication date: 2020 Apr 18

JOURNAL INFORMATION
==============================
NLM Title Abbreviation: Can Med Educ J
Journal ID: Can Med Educ J
NLM Journal ID: 101560935
Journal ID: CMEJ

Canadian Medical Education Journal

EISSN: 1923-1202
Publisher: University of Saskatchewan

ARTICLE INFORMATION
==============================
Subjects: Of 7–4

Does variability in overnight call schedules prior to the Internal Medicine clerkship exam influence student grades?

Nicholas Sequeira University of Toronto

Catherine Matolcsy University of Toronto

Edmund Lorens University of Toronto

Luke Devine University of Toronto

Electronic publication date: 2020 Apr 18

JOURNAL INFORMATION
==============================
NLM Title Abbreviation: Can Med Educ J
Journal ID: Can Med Educ J
NLM Journal ID: 101560935
Journal ID: CMEJ

Canadian Medical Education Journal

EISSN: 1923-1202
Publisher: University of Saskatchewan

ARTICLE INFORMATION
==============================
Subjects: Of 7–5

Perception of the Influence of a Humanities Curriculum on the Development of Patient-Centered skills in Family Medicine Residents

Nina Nguyen University of Ottawa

Andrea Zumrova University of Ottawa

Alan Ng University of Ottawa

Electronic publication date: 2020 Apr 18

JOURNAL INFORMATION
==============================
NLM Title Abbreviation: Can Med Educ J
Journal ID: Can Med Educ J
NLM Journal ID: 101560935
Journal ID: CMEJ

Canadian Medical Education Journal

EISSN: 1923-1202
Publisher: University of Saskatchewan

ARTICLE INFORMATION
==============================
Subjects: Of 7–6

New Graduate Health Professionals' Experiences with Employer Supports

Kevin Eva University of British Columbia

Teresa Green Vancouver Coastal Health

Electronic publication date: 2020 Apr 18

JOURNAL INFORMATION
==============================
NLM Title Abbreviation: Can Med Educ J
Journal ID: Can Med Educ J
NLM Journal ID: 101560935
Journal ID: CMEJ

Canadian Medical Education Journal

EISSN: 1923-1202
Publisher: University of Saskatchewan

ARTICLE INFORMATION
==============================
Subjects: Of 7–3

'A roller coaster of emotions': A phenomenological study into the lived experience of medical students emotions in simulation

Gerard Gormley Queen's University Belfast

Claudi Perez Medical Education Unit, Universidad Católica del Norte

Erik Driessen School of Health Professions Education, Maastricht University

Diana Dolmans School of Health Professions Education, Maastricht University

Electronic publication date: 2020 Apr 18

